# A compendium of transcription factor and Transcriptionally active protein coding gene families in cowpea (*Vigna unguiculata* L.)

**DOI:** 10.1186/s12864-017-4306-1

**Published:** 2017-11-22

**Authors:** Vikram A. Misra, Yu Wang, Michael P. Timko

**Affiliations:** 1 Department of Biology, University of Virginia, Gilmer Hall 044, Charlottesville, VA 22904 USA; 2 Center for Quantitative Sciences, Vanderbilt University, Nashville, TN 37232-6848 USA

**Keywords:** Cowpea, Common bean, Phylogenetic analysis, Soybean, Transcription factor

## Abstract

**Background:**

Cowpea (*Vigna unguiculata* (L.) Walp.) is the most important food and forage legume in the semi-arid tropics of sub-Saharan Africa where approximately 80% of worldwide production takes place primarily on low-input, subsistence farm sites. Among the major goals of cowpea breeding and improvement programs are the rapid manipulation of agronomic traits for seed size and quality and improved resistance to abiotic and biotic stresses to enhance productivity. Knowing the suite of transcription factors (TFs) and transcriptionally active proteins (TAPs) that control various critical plant cellular processes would contribute tremendously to these improvement aims.

**Results:**

We used a computational approach that employed three different predictive pipelines to data mine the cowpea genome and identified over 4400 genes representing 136 different TF and TAP families. We compare the information content of cowpea to two evolutionarily close species common bean (*Phaseolus vulgaris*), and soybean (*Glycine max*) to gauge the relative informational content. Our data indicate that correcting for genome size cowpea has fewer TF and TAP genes than common bean (4408 / 5291) and soybean (4408/ 11,065). Members of the GROWTH-REGULATING FACTOR (GRF) and Auxin/indole-3-acetic acid (Aux/IAA) gene families appear to be over-represented in the genome relative to common bean and soybean, whereas members of the MADS (Minichromosome maintenance deficient 1 (MCM1), AGAMOUS, DEFICIENS, and serum response factor (SRF)) and C2C2-YABBY appear to be under-represented. Analysis of the AP2-EREBP APETALA2-Ethylene Responsive Element Binding Protein (AP2-EREBP), NAC (NAM (no apical meristem), ATAF1, 2 (Arabidopsis transcription activation factor), CUC (cup-shaped cotyledon)), and WRKY families, known to be important in defense signaling, revealed changes and phylogenetic rearrangements relative to common bean and soybean that suggest these groups may have evolved different functions.

**Conclusions:**

The availability of detailed information on the coding capacity of the cowpea genome and in particular the various TF and TAP gene families will facilitate future comparative analysis and development of strategies for controlling growth, differentiation, and abiotic and biotic stress resistances of cowpea.

**Electronic supplementary material:**

The online version of this article (10.1186/s12864-017-4306-1) contains supplementary material, which is available to authorized users.

## Background

Cowpea (*Vigna unguiculata L. Walp*) is an important grain legume in the sub-tropics and the most important food and forage legume in sub-Saharan Africa [1, 2]. Estimates by the Food and Agriculture Organization (FAO) of the United Nations [3] indicate that 5.59 million metric tons of cowpea was produced worldwide, the majority of which (81%) is produced by low-input subsistence farmers in Western Africa [3–5], followed by Eastern (8.68%) and Central Africa (4.37%) [3]. In these regions cowpea grains are an important source of protein and carbohydrates [5, 6]. In addition to the fruit, leaves are also eaten [7], and cowpea stems are an effective fodder for livestock [8]. Moreover, cowpea can be used to restore nitrogen to soils [9], making it an effective companion crop to cereals [10, 11]. Furthermore, cowpea can withstand dry conditions and low quality soils relatively well [12].

Like all plants, cowpea faces a myriad of challenges from abiotic and biotic factors that constrain its growth and productivity [5, 6, 13–16]. Among the most significant stresses are attacks from root parasitic angiosperms [5], drought and increased soil salinity [17], which lead to significant or even total losses of yield [18–20]. Despite the social and economic importance of the crop, until recently genomic scale information was not available for cowpea putting its improvement at a disadvantage relative to other legumes such as soybean, common bean, and chickpea [21–25]. Initial attempts to capture genomic scale information [26] using reduced representation cloning and sequencing provided information on about 70% of the estimated 620 Megabase (Mb) genome, including information of transcription factors and resistance related genes. Recently, a draft genome sequence assembly providing 65× coverage has been reported by Muñoz-Amatriaín et al. (2017) [27] making possible a much more robust analysis of the informational content of this species.

Prior studies on the genomic contents of plants have tackled uncovering the content and complexity of transcription factors (TFs) and transcriptionally active proteins (TAPs, syn. Transcription associated proteins) present in the genome. Beginning with the first genome-scale analyses of TFs and TAPs in *Arabidopsis thaliana* almost two decades ago (Riechmann et al., 2000) [28], genome scale studies of these important regulatory molecules have appeared for a wide variety of plant species including rice (Gao et al., 2006) [29], poplar (Zhu et al., 2007) [30], soybean (Schmutz et al., 2010) [31] and other legumes (Richardt et al., 2007; Udvardi et al., 2007 [32, 33]) and tobacco (Rushton et al., 2008) [34].

Comparative information on TF and TAP content in genomes can be found in various databases based upon different discovery pipelines (e.g., PlantTAPDB (Plant Transcription Associated Protein Database) (Richardt et al., 2007) [32], PlnTFDB (Plant Transcription Factor Database) (Riano-Pachon et al., 2007; Perez-Rodriguez et al., 2010) [35, 36], PlantTFDB (Plant Transcription Factor Database) [37], GreenPhylDB (Rouard et al., 2011, 2014; http://www.greenphyl.org/cgi-bin/index.cgi) [38, 39], and PlantTFcat pipeline [40]). Some of these databases identify only TFs, while others include transcription regulators (TRs) and TAPs, and chromatin remodelers (CRs). In some cases, the pipelines do not include the range of all known TFs and TAPs. The most comprehensive data to date on the TF and TAP content of legumes can be found in the iTAK Plant Transcription Factor & Protein Kinase Identifier and Classifier database [41, 42], which contains TF and TAP content of 74 plant species, including the legumes *Medicago truncatula*, *Lotus japonicus*, chickpea, soybean, pigeon pea and common bean, but not cowpea. At present the only information on TFs for cowpea exists on the *Vigna unguiculata* Gene Expression Atlas (VuGEA) database [43], but this information relies solely on transcriptomic data.

Therefore, to address the lack of information available for this species, we have utilized the recently published draft genome assembly for cowpea [27] and applied existing and novel computation pipelines to create a comprehensive dataset of TFs and TAPs for cowpea. We also compared the genomic content of cowpea with two of its evolutionarily close relative legumes, common bean (*Phaseolus vulgaris*) and soybean (*Glycine max*). We also highlight information on several selected TF families in cowpea involved in stress responses and compare the content and phylogenetic organization of these families in cowpea to their counterparts in common bean.

## Results

### Identification of TFs and TAPs

A draft genome assembly has been generated that provides 67X coverage of the estimated 620 Mb cowpea genome [27]. The assembly includes 39 Gb of Illumina GAII (Genome Analyzer II) paired-end sequences (70–130 base), and ~250,000 gene-space sequences (GSSs) (average length of 609 nucleotides). About 97% of all previously reported cowpea expressed sequence tags (ESTs) can be found in the assembly by Basic Local Alignment and Search Tool Nucleotide (BLASTN) and a large proportion of the assembly is composed of scaffold sequences containing two or more overlapping contigs [27]. To identify genes encoding TFs and TAPs present in the draft cowpea genome assembly, we used three different identification pipelines: the PlantTFcat pipeline [40], the iTAK pipeline [41] and a novel pipeline developed for this study that uses a strict set of rules for gene family membership as defined by Lang et al. (2010) [44]. The latter pipeline is capable of identifying 111 TF and TAP families and is based on 223 rules (134 “mandatory” and 89 “forbidden”) focused on the presence/absence of specific domains in certain families. In this case, 124 domain hidden Markov models (HMMs) and 108 domains obtained from the Pfam protein family database were used to identify sequences as TFs and TAPs. This pipeline originally used the TavernaPBS software [45], which was made using the Taverna software [46] to identify and characterize TFs and TAPs on a PBS (Portable Batch System) cluster (Additional file 1). We adapted this pipeline into a Bash shell script that we developed to run on a SLURM (Simple Linux Utility for Resource Management) cluster.

Using these three approaches, we identified a total of 5460 TF- and TAP-encoding domains from 4416 sequences falling into 136 families. Multiple TF-encoding regions came from the same sequence in part due to the translation of TF-encoding transcript sequences to protein, which may have yielded different TFs on different reading frames. When sequences with multiple open reading frames (ORFs) are taken into account, these 5460 TFs come from 4416 sequences, which represent 7.26% of the 60,838 transcript genes (56,626 of which directly code for protein) in the 620 Mb of the cowpea genome. We also applied these same rules and pipelines to identify the TFs and TAPs in two evolutionarily close relatives, common bean and soybean. The number of TFs and TAPs found were 6468 and 13,419 sequences, respectively, for common bean and soybean. At 4416 TF-encoding sequences, cowpea has ~ 31.7% fewer TF/TAPs than common bean, which, like cowpea, is diploid, and only 32.9% of the content of soybean, a tetraploid legume (Additional file 2a). When only the genes and not the gene models are taken into account, the 4416 TF sequences together come from 4408 TF genes (Additional file 2b), and common bean and soybean have 5291 and 11,065 TF genes, respectively, making the number of cowpea TF/TAP genes 16.7% smaller than in common bean, and only 39.8% as many as in soybean (Additional file 2b). Thus, the number of TFs and TAPs in cowpea is relatively small for a diploid legume.

In order to assess the quality of our identification and classification approach we compared our results with selected publications in which detailed analyses had been carried out for TF/TAP families of other plant species. The combination of the three pipelines used in this study suggests that the TFs and TAPs in cowpea is relatively small in number of TFs and TAPs to other diploid plants, and that in terms of percentage of protein-coding genes, cowpea has a small proportion of TFs and TAPs. Compared to data from other databases, though, cowpea (and related legumes) has a relatively large number of TFs, although this could be due to differences between the pipelines used in other databases and the combination of three pipelines used in this study. The cowpea TF and TAP repertoires resulting from analyses using the PlantTFcat, iTAK and the TavernaPBS pipelines individually are shown in Additional file 3. According to data on the iTAK Plant Transcription Factor & Protein Kinase Identifier and Classifier Database [41, 42], the adzuki bean genome (*Vigna angularis*) [47], has 2755 total TF-encoding and TAP-encoding genes (2260 TFs and 495 TRs) across 92 families [42]; since adzuki bean has 26,857 genes, TFs and TAPs account for approximately 10.3% of protein-coding genes in adzuki bean. The common bean genome from Schmutz et al. (2014) [48], which consists of 27,197 genes, is found on iTAK to have 2779 TFs and TAPs (2314 TFs and 465 TRs), or 10.2% of genes, across 89 families [42]. *Medicago truncatula* genome v4.0v1 [49], which has 50,894 genes, is found on iTAK to have 3670 TFs and TRs (2948 TFs and 722 TRs), or 7.2% of genes, across 89 families [42].

Others who studied the repertoire of cowpea TFs, TRs and CRs did not find data as fully comprehensive as the data found in this study. PlantTFDB v4.0 [37] contains only 488 TFs from 48 families in cowpea [37]. The VuGEA database [43], which used the PlantTFcat pipeline to identify TFs, TRs and CRs in the cowpea transcriptome, found 2485 TFs, TRs and CRs out of 24,866 cowpea unigenes (10% of unigenes). Thus, this study represents the most comprehensive study yet on cowpea TFs and TAPs.

Here, it must be noted that PlantTFcat, iTAK and the TavernaPBS pipeline used in this study were used to analyze the cowpea protein assembly, as well as the cowpea transcripts assembly. For comparison purposes, all three pipelines were also used to analyze the raw cowpea assembly. According to the PlantTFcat pipeline, the largest TF families in the raw cowpea genome assembly are Zinc-finger, CCHC-type (CCHC(Zn)) (2548), C2H2 (702) and WD-40-like (449) (Additional file 3). According to the iTAK pipeline, the largest three families in the raw genome are Myeloblastosis-related (MYB-related) (249), WRKY (175) andAP2/ERF-ERF (syn. ERF, ethylene response factor) (173). According to the TavernaPBS pipeline, MYB-related (396) and WRKY (302) are the second and third largest families behind B3 (known in the TavernaPBS pipeline as ABI3/VP1 (Abscisic Acid Insensitive 3 / Viviparous1)) (409). Here, it must be noted that the raw cowpea genome statistics on TF families is significantly different from those for the cowpea protein and transcript assemblies, with some families that are under-represented in the raw cowpea genome as opposed to the protein and transcript assemblies (e.g., ARF (Auxin Response Factor)) (Additional file 2), and other families being represented in the raw cowpea genome and not found in protein and transcript assemblies (e.g., Rel (Relish)). The presence of TF families in the raw cowpea genome and not the protein and transcript families could be an effect of the MAKER [50] and AUGUSTUS [51, 52] methods of annotation used for the cowpea genome v0.03 (see Materials and Methods). Due to the un-curated nature of the raw cowpea genome assembly, the TF families only found in the raw cowpea genome are not included in the TF statistics in Additional file 2.

In this study, several families were found that were not present in VuGEA, due to the use of iTAK and the TavernaPBS pipeline in this study. Such families include the NF-X1 (nuclear factor X-box binding 1), NF-YC (nuclear factor Y subunit C) (syn. CCAAT-HAP5 (CCAAT motif, heme-associated protein 5), SOH1 (suppressor of hyper-recombination 1), Rel, RF-X (regulatory factor X) and zn-clus (Zn(2)-Cys(6) binuclear cluster domain). In the cowpea protein and transcript assemblies, the NF-X1 (1 member), CCAAT-HAP5 (16), and SOH1 (1) are represented. In the raw cowpea genome assembly, members of four families were found that were not found in the cowpea protein or transcript assemblies. These families were JmjC-ARID (Jumonji C-terminal, AT-Rich Interaction Domain) (1), Rel (40), RF-X (40) and zn-clus (1).

It must be noted that in VuGEA, 3 cowpea transcripts were reported as belonging to the family ABTB (Ankyrin Broad Complex, tramtrack and bric a brac) (a sub-family of TRAF (Tumor necrosis factor receptor-associated factor)), and 2 others as members of CW-Zn-B3_VAL (CW-like zinc finger, B3, VP1/ABI3-Like) [53]. None of the pipelines in this study have found members of either family in cowpea, but our pipelines have found that 5 ABTB family members and 7 members of the CW-Zn-B3_VAL family exist in common bean. Selected common bean ABTB and CW-Zn-B3_VAL nucleotide sequences were used as queries against the raw cowpea v0.03 assembly in a FASTA search (FASTA version 36.3.8e [54]; E-value 10e-3). The FASTA search yielded 36 raw cowpea sequences that were homologous to ABTB and 28 raw sequences homologous to CW-Zn-B3_VAL (E-value < 10e-3).

**Fig. 1:**
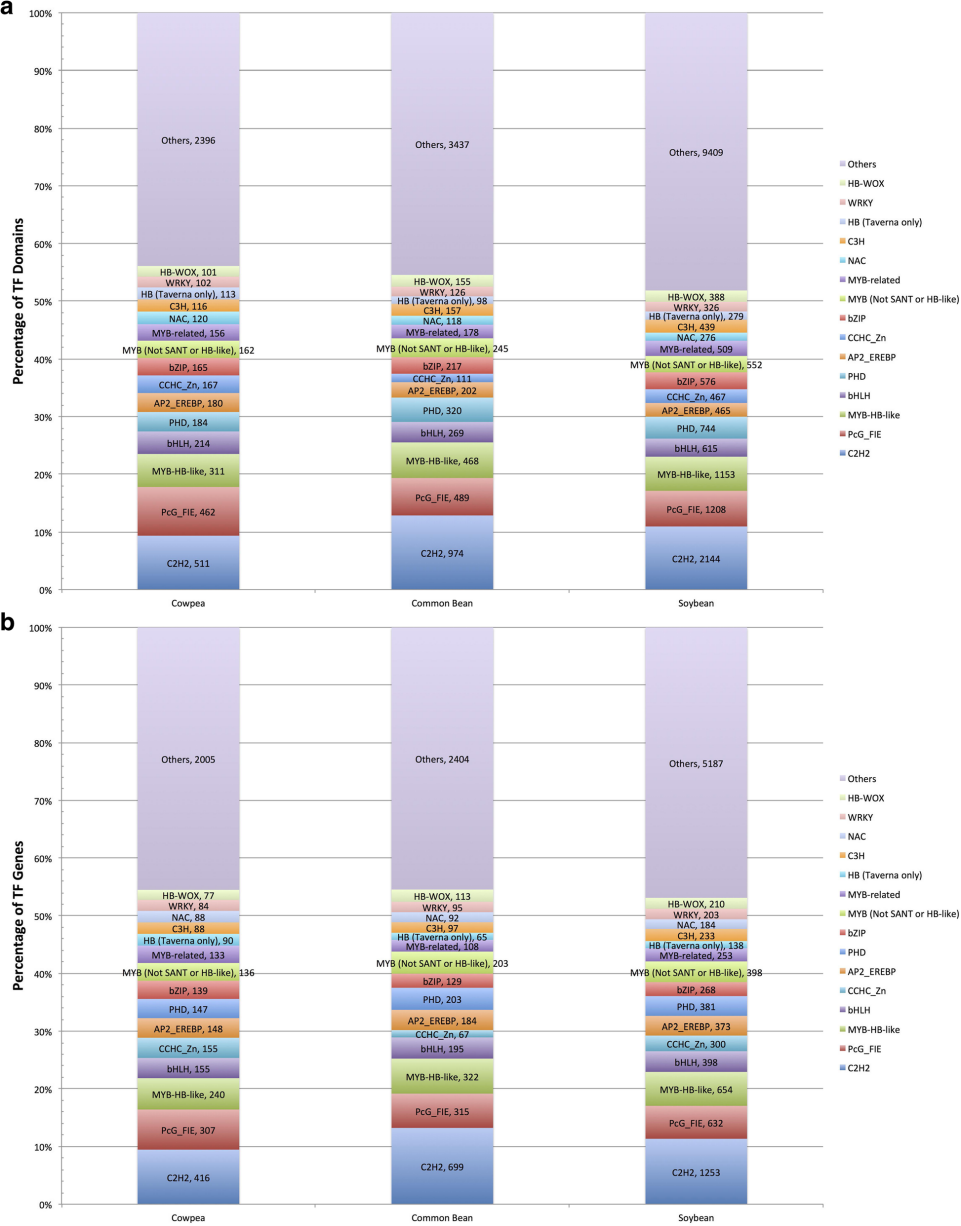
The largest cowpea TF and TAP families, and their sizes in common bean and soybean. Domains from all gene models are counted in (**a**) whereas genes (not counting gene models) are counted in (**b**). The largest fifteen families in cowpea account for approximately 56% of the cowpea TF, TR and TAP domains (and 54.5% of genes), whereas in soybean, these same families account for almost 52% of domains (and 53.1% of genes). Moreover, the over-representation of families such as PcG_FIE and the under-representation of families such as C2H2 in cowpea compared to common bean and soybean is shown

When the cowpea protein and transcript assemblies are taken into account, the largest TF/TAP family in cowpea is C2H2 (511, 11.57% of cowpea repertoire), followed by Polycomb Group Fertilization-Independent Endosperm (PcG_FIE) (i.e., WD-40) (462, 10.46%), MYB-HB-like (MYB Homeobox like) (311, 7.04%), and basic helix-loop-helix (bHLH) (214, 4.85%) (Fig. 1a, Additional file 2a). Similar to cowpea, the three largest TF families in common bean are C2H2 (974, 15.06% of bean TF repertoire), PcG_FIE (489, 7.56%), and MYB-HB-like (468, 7.24%). This was also true of soybean, with C2H2 (2144, 15.98% of soy TF repertoire), followed by PcG_FIE (1208, 9.00%), and MYB-HB-like (1153, 8.59%) being the three largest families. It is worth noting, though, that of these families, cowpea is under-represented in C2H2 and MYB-HB-like, and over-represented in PcG_FIE.

When only the genes and not the gene models are taken into account, the largest cowpea TF/TAP family is C2H2 (416, 9.44%), PcG_FIE (307, 6.96%), MYB-HB-like (240, 5.44%), bHLH and CCHC_Zn (155 each, 3.52% each) (Fig. 1b, Additional file 2b). For both common bean and soybean, C2H2 is the largest TF family (699 genes in common bean, 1253 genes in soybean). However, in common bean and soybean, unlike in cowpea, MYB-HB-like is the second largest (322 in common bean and 654 in soybean), and not the third largest family (which is PcG_FIE; 315 in common bean and 632 in soybean) (Additional file 2b).

Based on our prediction, Dicer is not represented in cowpea, but is represented in common bean and soybean. According to that same pipeline, CCAAT-Down regulator of transcription 1 (CCAAT-Dr1), Runt and transcriptional enhancer activator (TEA) are represented in cowpea (with each having one sequence), but not in common bean.

In determining whether certain cowpea TF families were over- or under-represented in relation to common bean and soybean, we compared cowpea TF families to common bean and soybean with respect to percentage of the respective TF repertoires. In order to determine whether or not the over- or under-representation was affected by gene number, we used two other criteria to compare cowpea TF repertoires to those of common bean and soybean: 1) raw numbers of TFs, and 2) percent of total protein-encoding transcript sequences. Over- or under-representation of a certain TF family in cowpea is depicted as a ratio; for example, the number of members of a TF family in cowpea divided by the number of members of that same TF family in common bean or soybean. If the ratio was between 0.9 and 1.1 for a certain family, that family was determined to be neither over- nor under-represented in cowpea. If the ratio exceeded 1.1, that family was determined to be over-represented in cowpea. If such ratios were smaller than 0.9, that family was under-represented in cowpea. The results for the comparisons between cowpea and common bean and soybean TF families based on these ratios are shown in Additional files 2 and 4.

Here it is important to note that common bean has 36,995 protein-coding transcripts and a genome size of 587 Mb [34], and that soybean has 88,647 protein-coding transcripts and a genome size of approximately 978.5 Mb [55]. For the statistics represented in Additional files 2 and 4, because both protein and transcripts are studied, and because in cowpea the protein coding sequences (56,626) are a subset of the set of transcripts (60,838), the percentage of total genes is represented by percentage of protein-coding transcripts.

When each cowpea TF family was compared to its counterparts in common bean and soybean with respect to percentage of their respective TF/TAP repertoires, common bean and soybean showed significant difference from cowpea (Additional files 2 and 4a).

When cowpea sequences (with all gene models accounted for) were compared to the diploid common bean on the basis of percentage of TF repertoire, cowpea was found to have 51 families under-represented, 31 families similarly proportioned, and 50 TF families over-represented compared to common bean. Also on the basis of percentage of TF repertoire, when only genes and not gene models are taken into account, results are similar to when gene models are taken into account. Cowpea, when compared to common bean, is under-represented in 51 families, similarly represented in 26 families, and over-represented in 55 families.

These results suggest that despite both cowpea and common bean being diploid legumes, that the cowpea TF repertoire is significantly different from that of common bean.

When cowpea sequences (with all gene models) were compared to the tetraploid soybean on the basis of percentage of TF repertoire, cowpea was found to have 29 TF families over-represented, 20 families similarly proportioned, and 87 TF families under-represented in terms of percentage of TF repertoire. While this result may be expected due to cowpea being diploid and soybean being tetraploid, it is important to note the presence of a significant proportion of cowpea TF families that were over-represented compared to both common bean and soybean. Of the 50 cowpea TF families that were over-represented compared to common bean, 21 were over-represented and 13 were similarly proportioned compared to their counterparts in soybean. When only genes are taken into account, compared to soybean, cowpea is under-represented in 48 families, similarly represented in 33 families, and over-represented in 55 families. Of the 55 TF families that were over-represented in common bean, 42 were over-represented and 4 were similarly proportioned compared to their counterparts in soybean. This presence of these families shows that the cowpea TF repertoire is unique in composition (Additional file 2).

Moreover, when each cowpea TF family was compared to its counterparts in common bean and soybean with respect to raw number of TFs, all but five of the cowpea TF families were found to be under-represented compared to soybean, while 15 cowpea TF families were over-represented, 19 families were similarly proportioned, and 98 were under-represented compared to common bean (Additional file 4b). This is expected since cowpea and common bean are diploid and soybean is tetraploid.

When the raw number of TFs and TAPs in cowpea and soybean were compared in such a way that each number of TFs in soybean was halved to provide a comparison that accounted for the tetraploidy of soybean and the diploidy of cowpea, cowpea was found to have 8 TF families that were over-represented compared to soybean, 7 families similarly proportioned to their counterparts in soybean, and the remaining 121 cowpea TF families were under-represented compared to soybean (Additional file 2). Here it is important to note that the number of soybean TF and TAP sequences divided in half is 6710, which is similar to the number of TFs in common bean (6468), while cowpea has 4416 TF and TAP sequences. Thus a comparison of cowpea to common bean and soybean can most accurately be based on percentage of their respective TF repertoires.

When each cowpea TF family was compared to its counterparts in common bean and soybean with respect to percentage of total protein genes, cowpea was, compared to common bean and soybean, under-represented in almost all TF families (Additional file 4c). This is consistent with expectations because in cowpea, TFs comprise 7.26% of all protein coding genes, which is a small percentage compared to common bean (17.5%) and soybean (15.1%) (Additional file 2).

These results suggest that the significant differences between cowpea TF families and their counterparts in fellow legumes common bean and soybean in terms of percentage of TF repertoire are not significantly affected by differences in gene number or genome size. For this reason, in all statistical analyses in which only the genes and not gene models are counted, any comparison between cowpea, common bean and soybean are done in terms of percentage of TF repertoire. Cowpea TF families whose size and complexity differ with respect to common bean and soybean have been identified (Additional file 2). For example, when all gene models are counted, cowpea TF families that were over-represented in cowpea compared to common bean are PcG_FIE (462), followed by CCHC(Zn) (167) and NAC (120). The largest cowpea TF families that were similarly proportioned to their counterparts in common bean are bHLH (214), AP2-EREBP (180) and basic leucine zipper domain (bZIP) (165). The C2H2 (511), MYB-HB-like (311) and Plant Homeodomain (PHD) (184) families are the largest cowpea TF families that are under-represented with respect to common bean. Similarly, in a comparison of TF families between cowpea and soybean based on percentage of respective TF repertoires, PcG_FIE (462), followed by AP2-EREBP (180) and NAC (120) were the largest cowpea TF families that were over-represented compared to soybean. Of the cowpea TF families that are similarly proportioned to their counterparts in soybean, bHLH (214), CCHC(Zn) (167) and MYB-related (156) are the largest. C2H2 (511), MYB-HB-like (311) and PHD (184) are the largest cowpea TF families to be under-represented compared to soybean (Additional file 2a).

When only genes and not gene models are taken into account, the largest cowpea families over-represented compared to common bean are PcG_FIE (307), CCHC_Zn (155) and bZIP (139). The largest cowpea families similarly proportioned to their counterparts in common bean are bHLH (155), AP2_EREBP (148), and C3H (88). The largest cowpea families under-represented compared to common bean are C2H2 (416), MYB-HB-like (240) and PHD (147). The largest cowpea families over-represented compared to soybean are PcG_FIE (307), CCHC_Zn (155), and bZIP (139). The largest cowpea families similarly proportioned to their counterparts in soybean are MYB-HB-like (240), bHLH (155) and AP2-EREBP (148). The largest cowpea families under-represented compared to soybean are C2H2 (416), MYB (136) and CCAAT_HAP3 (62) (Additional file 2b).

Interestingly, according to the TavernaPBS pipeline, some families, such as CCAAT-HAP3 (CCAAT motif, heme-associated protein 3) (syn. NF-YB (nuclear factor Y subunit B)), are fewer in number than expected (Additional file 2), especially given that other organisms were found to have several CCAAT-HAP3 sequences, like the fifteen CCAAT-HAP3 sequences found in tobacco [34]. This could be due to an artifact in the TavernaPBS pipeline used in this study to identify and classify TFs: when the pipeline found a sequence with required domains for two or more TF families, the pipeline would sometimes not assign a TF family to the sequence. For example, a sequence with similarity to an NF-YB domain, a required domain of the CCAAT-HAP3 TF family, may have also been found to have similarity to an NF-YC domain, a domain required for CCAAT-HAP5 [30]. In this situation, the pipeline did not classify the sequence into any particular TF family. One possible future approach to improving the TavernaPBS pipeline used in this study is to assign a sequence to a family based on the TF domain to which it has the strongest similarity.

In this study, we compensated for the artifacts in the TavernaPBS pipeline by incorporating statistics from a search for cowpea TFs using the PlantTFcat [40] and iTAK pipelines [41]. These pipelines yielded statistics for CCAAT-HAP3 and CCAAT-HAP5 that were closer to expected for a diploid legume (Additional files 2 and 3).

### Phylogenetic analysis of TF families

The AP2-EREBP, NAC and WRKY families were chosen for deeper analysis due to their involvement with defense response in plants [25, 56, 57] and because each family has easily recognizable conserved domains [56, 58–60]. After a multiple sequence alignment using MAFFT (Multiple Alignment using Fast Fourier Transform) L-ins-i version 7.245 [61], a phylogenetic analysis using maximum likelihood in RAxML (Randomized Axelerated Maximum Likelihood) [62] with 100 bootstrap replicates was performed on each of the three families. In these phylogenetic analyses, amino acid sequences, and therefore all gene models, are used.

#### AP2-EREBP family

The AP2-EREBP superfamily first described by Ohme-Takagi & Shinshi (1995) [58] in tobacco controls multiple processes in plants from development [63] to defense against abiotic and biotic stresses [64, 65]. The AP2-EREBP superfamily consists of three families: ERF, Related to ABI3/VP1 (RAV) and AP2 [66, 67]. The ERF family has distinguishing conserved motifs such as an N-terminal AEIRD motif and a WLG [66]. The RAV family has an AP2 and a B3 domain [67]. The AP2 family usually has two AP2 domains [66]. The first (N-terminal) domain, known as the R1 repeat, usually has a YEAH or WESHI at the 5′ end and a YDRAA or LAALKY at the 3′ end, whereas the second (C-terminal) domain, known as the R2 repeat, has a WQAR or WEAR at the 5′ end and a NAVT or YDIAAI at the 3′ end [66, 68]. The AP2 family also has a conserved YLG instead of the WLG and AEIRD found in ERFs [66, 68].

**Fig. 2:**
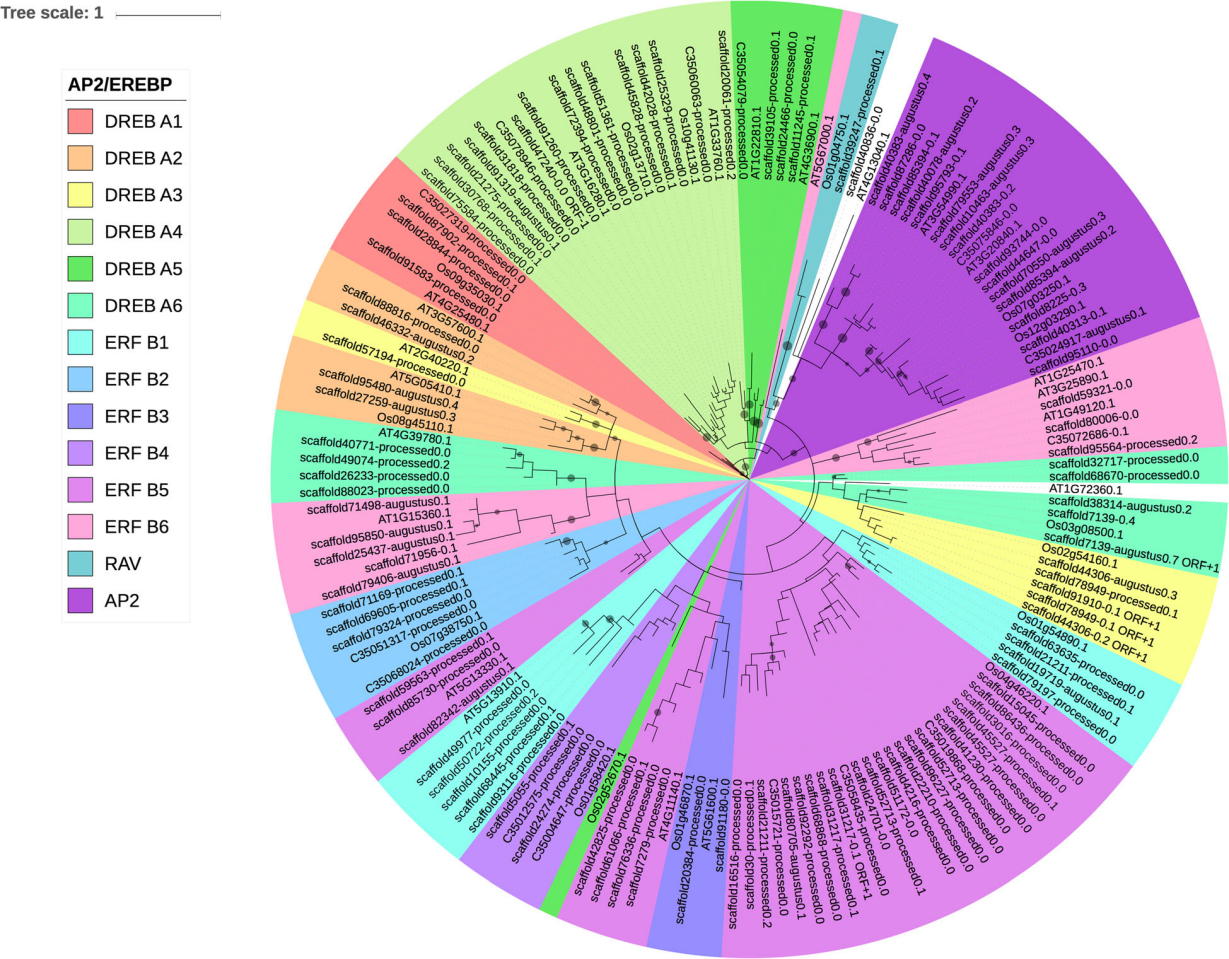
Cowpea AP2-EREBP genes according to the DREB A1-A6 and ERF B1-B6 grouping. This grouping follows the classification schemes of Dietz et al. (2010) [69] and Sharoni et al. (2011) [70]. The trees were generated using RAxML [62] with 100 bootstrap values with the optimal amino acid substitution model automatically chosen in RAxML (i.e., the PROTGAMMAAUTO option). The circles on the branches are bootstrap support values from 50 to 100, with the largest circles representing the greatest bootstrap support

**Fig. 3:**
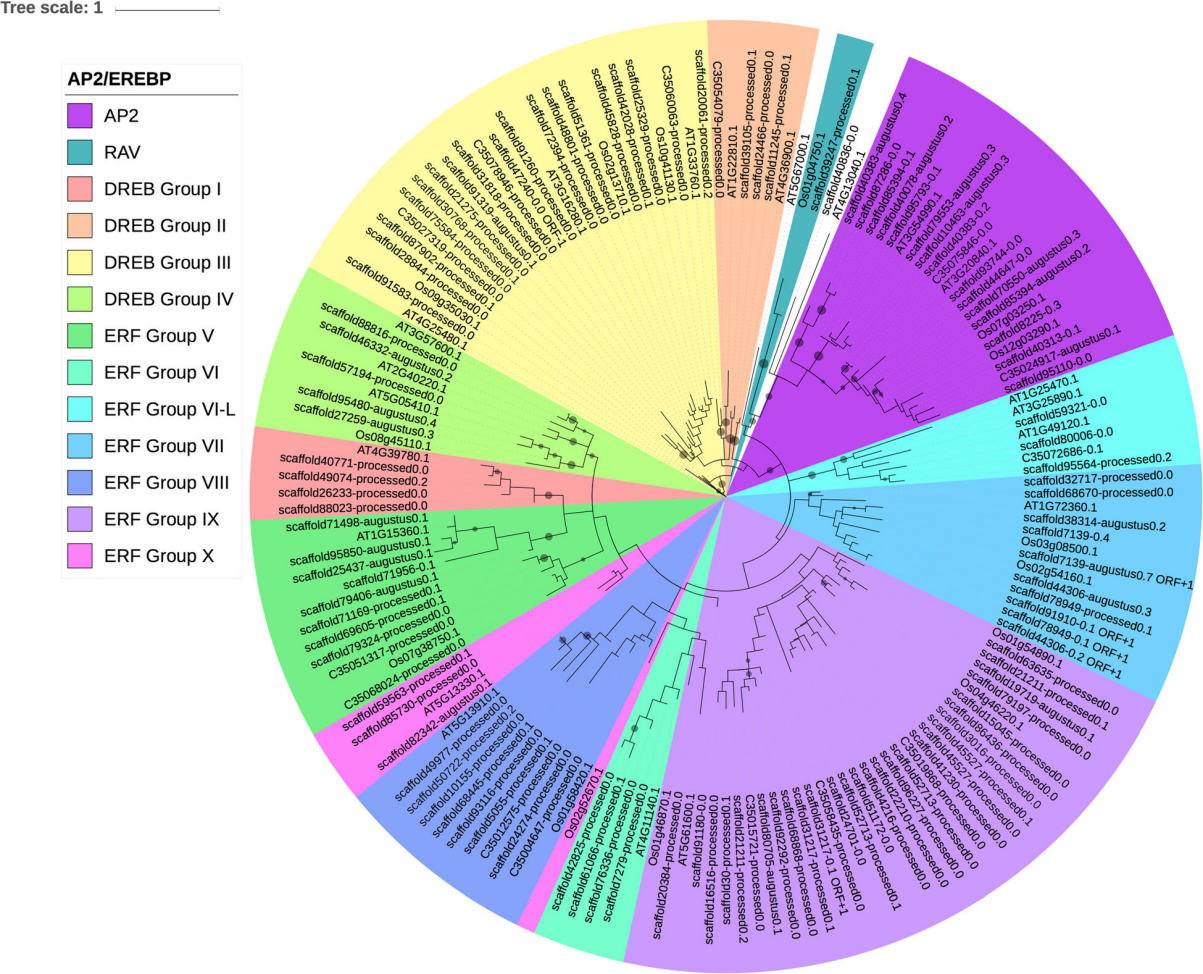
Cowpea AP2-EREBP genes according to the grouping into groups I to X. This grouping is used in Nakano et al. (2006) [64]. The trees were generated using RAxML [62] with 100 bootstrap values with the optimal amino acid substitution model automatically chosen in RAxML (i.e., the PROTGAMMAAUTO option). The circles on the branches are bootstrap support values from 50 to 100, with the largest circles representing the greatest bootstrap support

The *Arabidopsis* and rice sequences were chosen in order to facilitate the classification of AP2-EREBP sequences into clades. The phylogenetic tree for the AP2-EREBP family (shown in Figs. 2 and 3) include 122 AP2-EREBP cowpea sequences along with the 22 *Arabidopsis* and 15 rice sequences representative of each clade of the AP2-EREBP superfamily. When cowpea ERFs were grouped according to the grouping scheme in Dietz et al. (2010) [69], namely groups Dehydration-responsive element (DRE)-binding (DREB) A1-A6 and ERF B1-B6, the Dietz et al. (2010) [69] and Sharoni et al. (2011) [70] classification was found not to be entirely consistent with the classification in Nakano et al. (2006) [64]. For example, group II in Nakano et al. (2006) [64] is supposed to contain only members of the DREB A-4 clade. Instead, group II sequences contained DREB-A4 and DREB-A5 sequences (Figs. 2 and 3). Moreover, group V in Nakano et al. (2006) [64] contained members of ERF B-2 and ERF B-6 from Dietz et al. (2010) [69] and Sharoni et al. (2011) [70] (Figs. 2 and 3). According to Nakano et al. (2006) [64], group V ERFs should only contain sequences from the ERF B-6 clade. Here it must be noted that Nakano et al. (2006) [64] divided *Arabidopsis* AP2-EREBP TFs into 12 clades and AP2-EREBP members in rice into 15 clades, and that the clades in this study are the 12 clades found in *Arabidopsis*. 11 of these clades are held in common between monocots and dicots [64].

#### NAC family

**Fig. 4:**
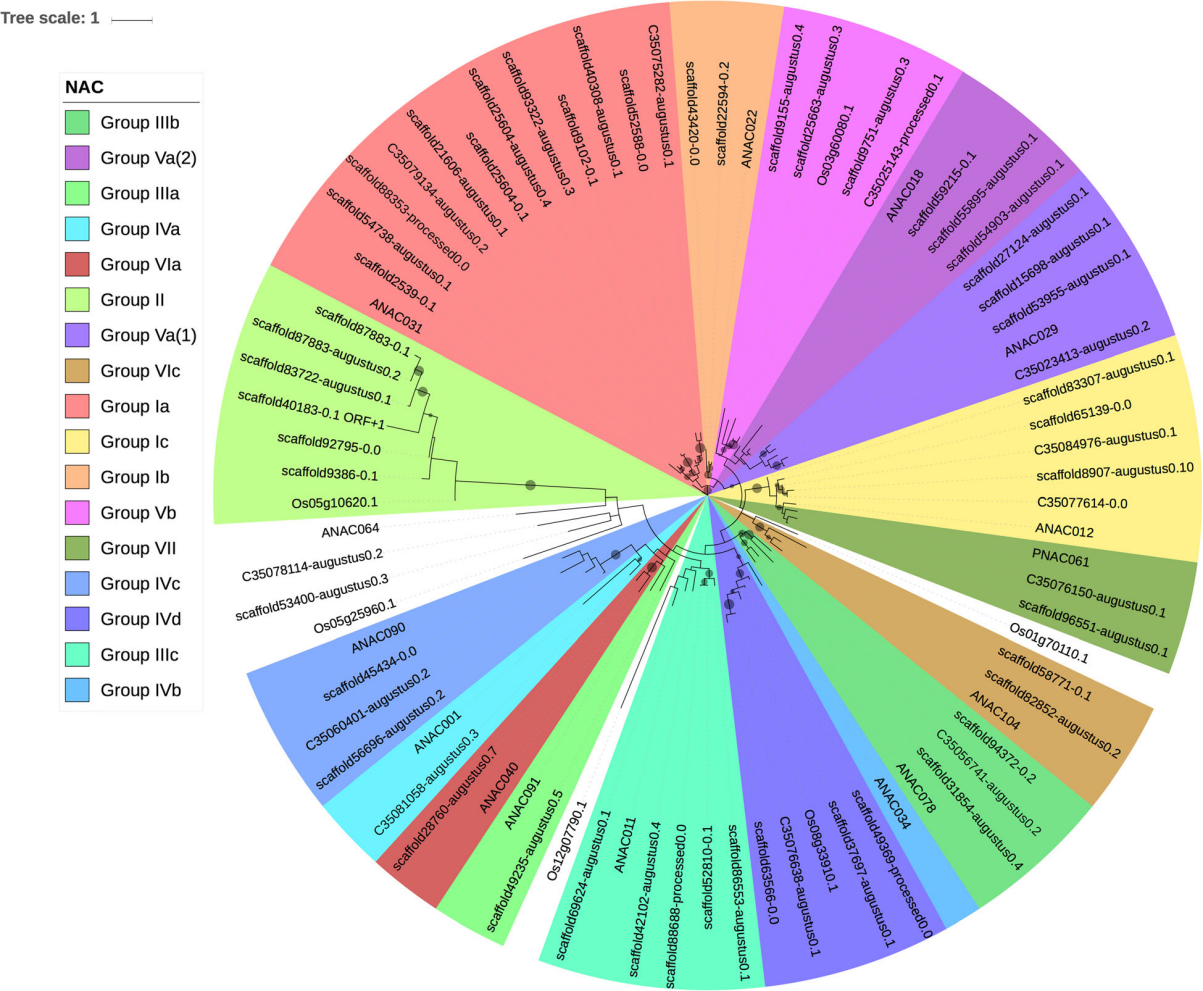
Phylogenetic tree of the NAC sequences. The tree was calculated based on NAC domain sequences of cowpea, as well as the representative NAC domains used in the methodology of Zhu et al. (2012) [71]. The tree was generated using RAxML [62] with 100 bootstrap values with the optimal amino acid substitution model automatically chosen in RAxML (i.e., the PROTGAMMAAUTO option). The circles on the branches are bootstrap support values from 50 to 100, with the largest circles representing the greatest bootstrap support

Methods of classifying NAC TFs vary greatly with one classification scheme by Ooka et al. (2003) [71] separating the family into two broad categories (I and II) with category I consisting of 15 groups named after individual members (e.g., *Arabidopsis* transcription activation factor 2 (ATAF2), Senescence Upregulated 5 (Senu5)) and category II containing 3 groups (e.g., ONAC003 (*Oryza sativa* NAC 003)). Rushton et al. (2008) [34] simplified the organization defining six clades (1 through 6) in most species and 3 clades unique to Solanaceae. Zhu et al. (2012) [72] classify NACs into ten numbered groups (I-X), some of which contain several subgroups. According to the classification scheme different species will have NAC families with different groups, depending on whether the plant is a monocot, dicot, moss or lycophyte, with dicots usually having Groups Ia-c, II, IIIa-c, IVa-d, Va(1), Va(2), Vb, VIa, VIc, VII, and VIII. All of these groups, with the exception of groups IVb and VIII, are found in the cowpea NAC family (Fig. 4).

Some more subtle differences exist between the cowpea NAC family organization found here and that reported by Zhu et al. (2012) [72] for dicots. First, Group II in cowpea does not sit on the same location within the NAC tree (i.e., between Groups I and III) as reported in Zhu et al. (2012) [72]. Second, Group VIa in cowpea groups next to IIIa, which is consistent with the Bayesian tree in Zhu et al. (2012) [72], but not the maximum likelihood tree from that same paper.

#### The WRKY family

The WRKY TF family is characterized by a conserved N-terminal WRKYGQK motif and a motif resembling a zinc finger [59, 73]. Variations in the conserved parts of the WRKY domain allow separation into three major groups (Groups I-III) [59]. Group I WRKY TFs have two WRKY domains, with their C-terminal domains being functionally distinct from the N-terminal domains [74]. Group II is the most variable group in terms of amino acid sequence, with five subgroups designated IIa –IIe [59, 60, 74]. Group III WRKYs differ in zinc finger structure from group-I and -II WRKY TFs; a group III WRKY zinc finger has a C_2_-HC structure, as opposed to the C_2_-H_2_ in the other two WRKY groups [59, 60].

**Fig. 5:**
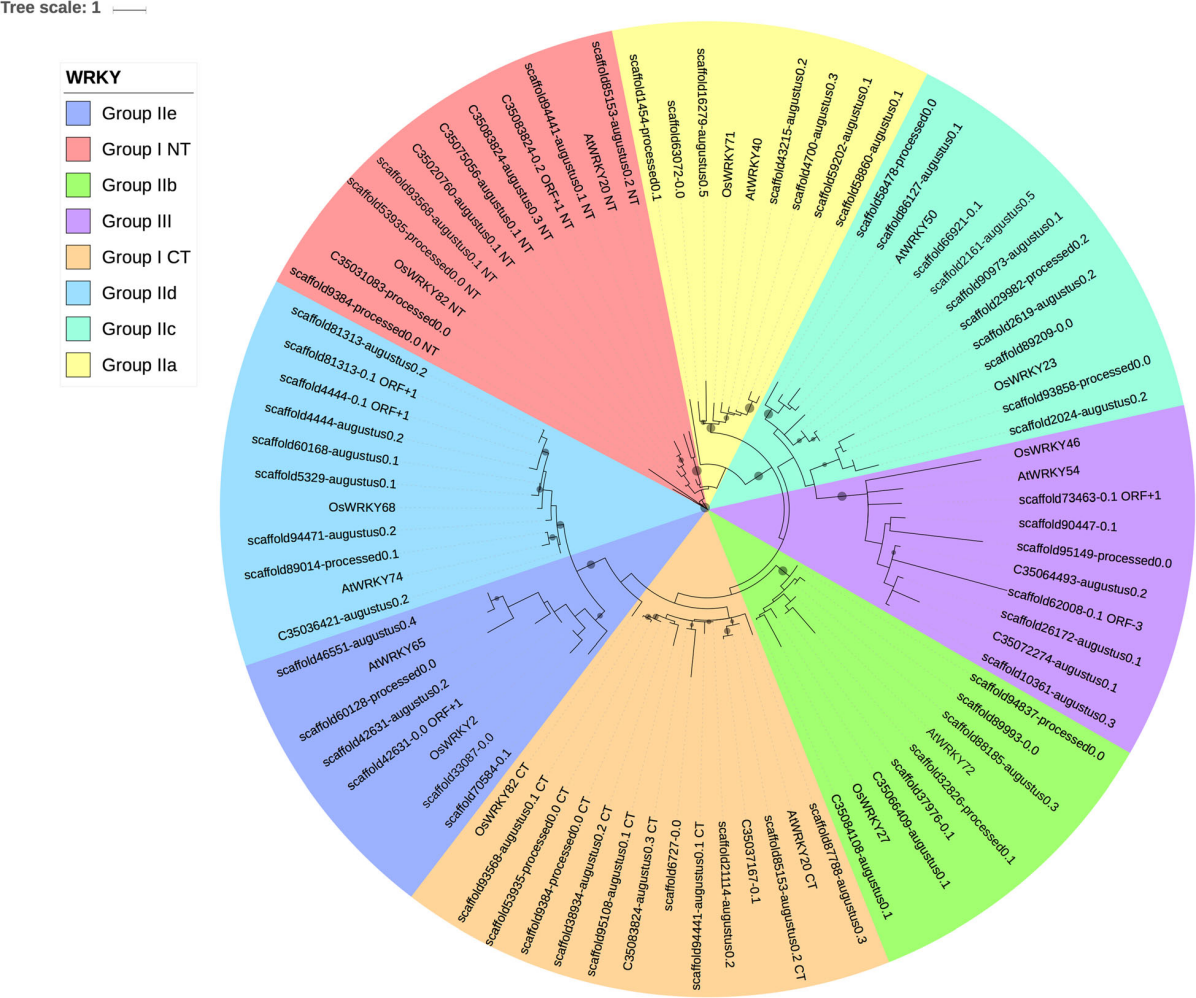
Phylogenetic tree of the cowpea WRKY genes. The tree was calculated based on WRKY domain sequences of cowpea, as well as selected representative *Arabidopsis* and rice WRKY domains; the selection of Arabidopsis and rice WRKY sequences was based on the methodology of Li et al. (2012) [73]. This tree was generated using RAxML [62] with 100 bootstrap values with the optimal amino acid substitution model automatically chosen in RAxML (i.e., the PROTGAMMAAUTO option). The circles on the branches are bootstrap support values from 50 to 100, with the largest circles representing the greatest bootstrap support

The relationship among the three groups of WRKY TFs can also be seen in the phylogenetic clustering of family members as depicted in Fig. 5. Notable about the tree are the following. Subgroups IId and IIe cluster together, similar to what was reported initially by Timko et al. (2008) [26] for the family. However, unlike the early phylogeny [26], subgroup IIb does not split into two distinct clades. The most likely explanation is that the earlier study had incomplete sequence data and the split was an artifact in the ClustalW alignment caused by truncated WRKY domains. This split did not happen in this study, probably because the alignment used in this study was based on MAFFT L-ins-i [61]. In addition, the sequences in this study were scaffold sequences, which were likely to contain two or more GSR or EST sequences, and hence would be more likely to have a complete WRKY domain.

### Comparison of cowpea and common bean TF families

**Fig. 6:**
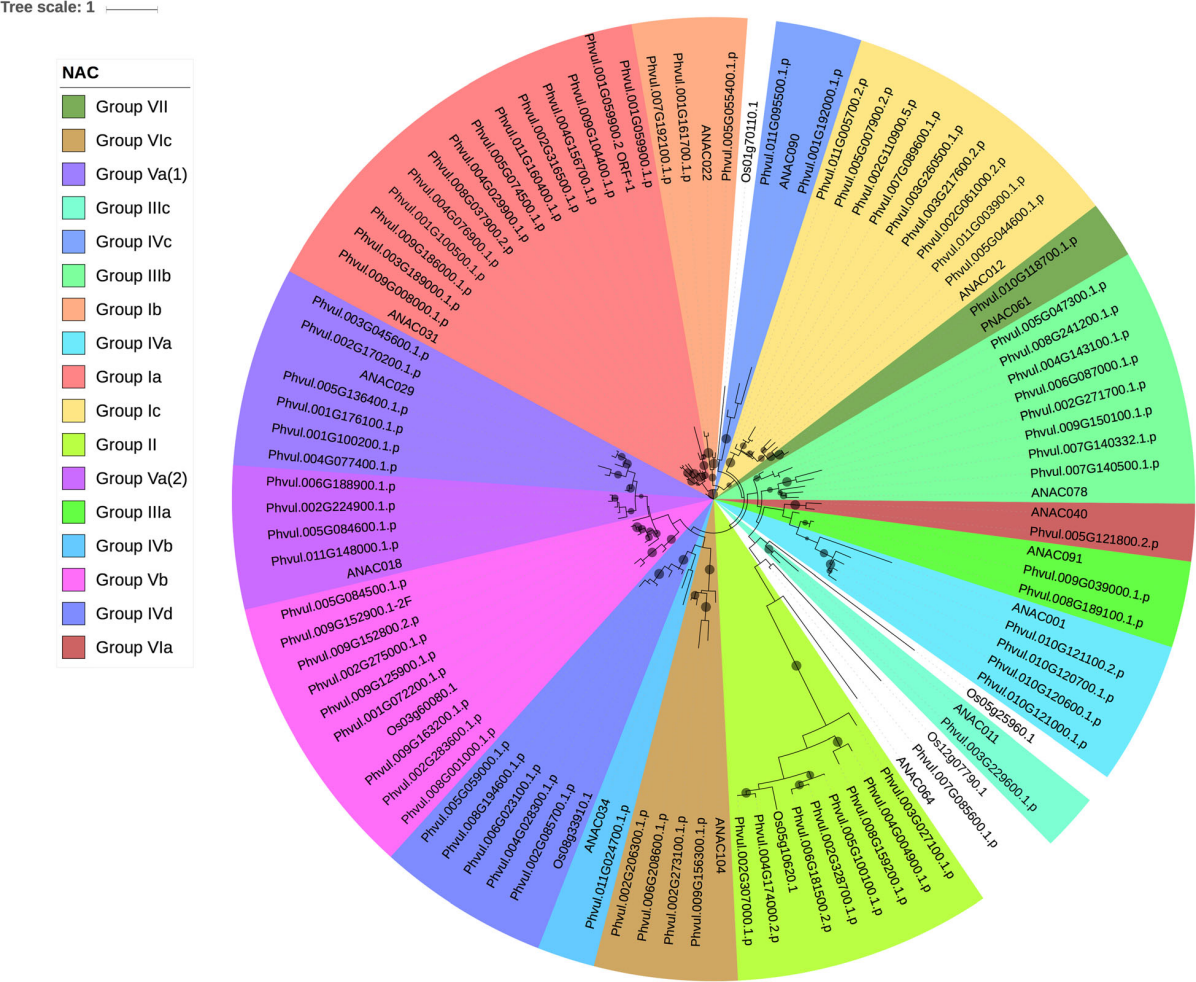
Phylogenetic tree of common bean NAC sequences. This tree was generated using RAxML [62] with 100 bootstrap values with the optimal amino acid substitution model automatically chosen in RAxML (i.e., the PROTGAMMAAUTO option). The tree was calculated based on NAC domain sequences of cowpea, as well as the representative NAC domains used in the methodology of Zhu et al. (2012) [71]. The circles on the branches are bootstrap support values from 50 to 100, with the largest circles representing the greatest bootstrap support

**Fig. 7:**
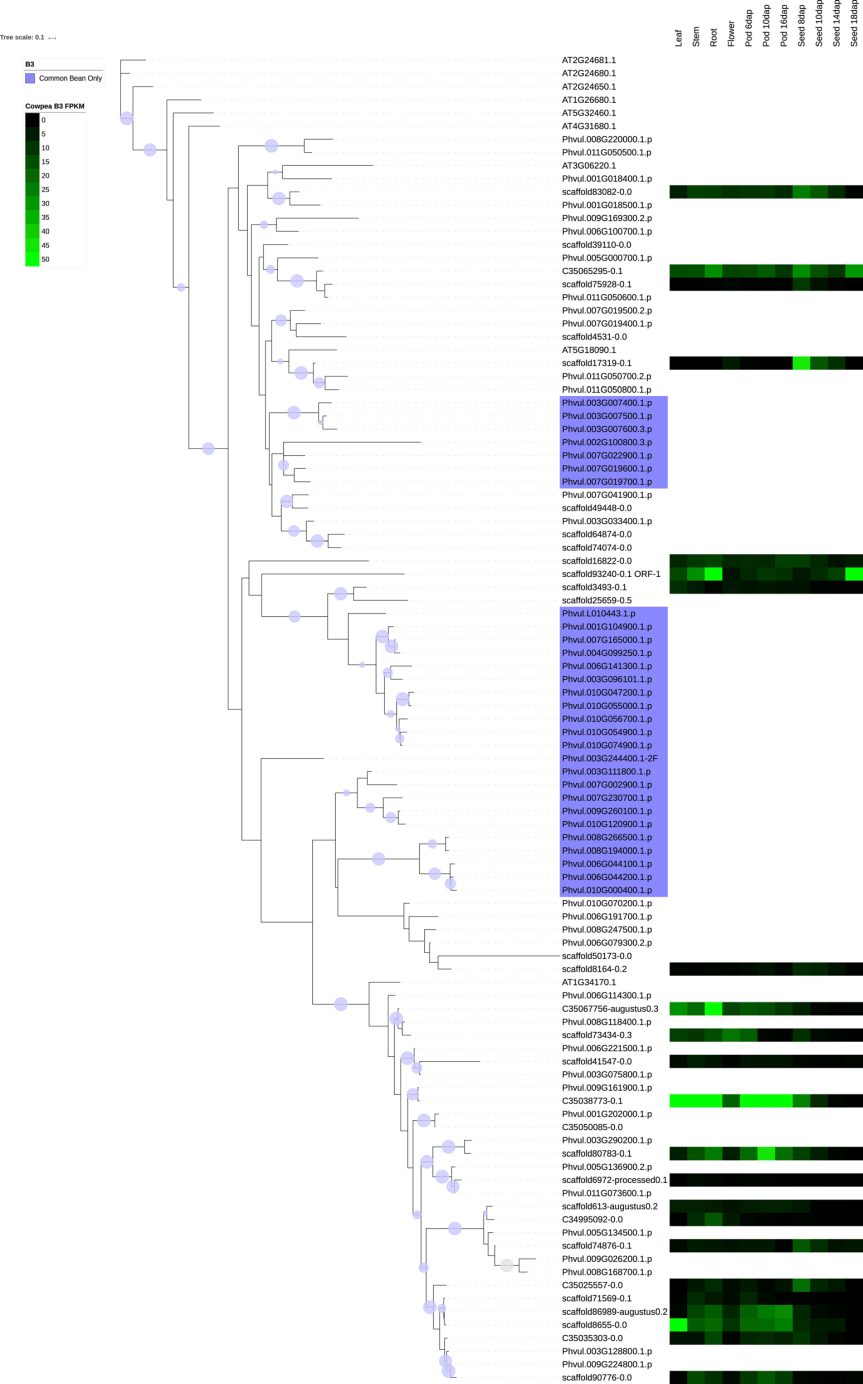
Cowpea and common bean B3 sequences, with predictive heatmaps for cowpea TFs based on FPKM expression values from cowpea transcriptome data on VuGEA [43]. This tree was generated using RAxML [62] with 100 bootstrap values with the optimal amino acid substitution model automatically chosen in RAxML (i.e., the PROTGAMMAAUTO option). Sequences starting with “C3” or “scaffold” are cowpea sequences, while sequences starting with “Phvul” (*Phaseolus vulgaris*) are from common bean. The circles on the branches are bootstrap support values from 50 to 100, with the largest circles representing the greatest bootstrap support

**Fig. 8:**
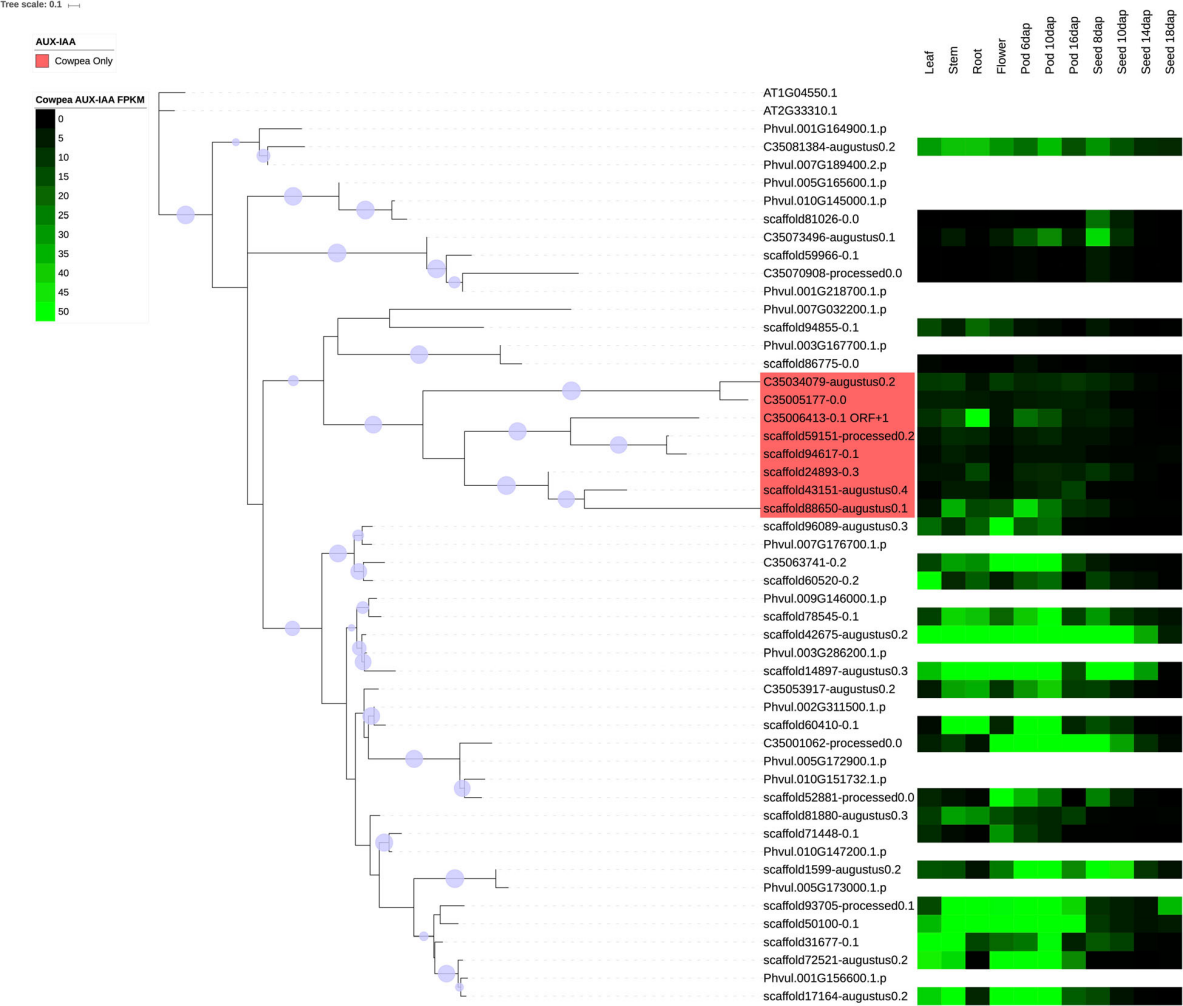
Cowpea and common bean AUX_IAA sequences, with predictive heatmaps for cowpea TFs based on FPKM expression values from cowpea transcriptome data on VuGEA [43]. This tree was generated using RAxML [62] with 100 bootstrap values with the optimal amino acid substitution model automatically chosen in RAxML (i.e., the PROTGAMMAAUTO option). Sequences starting with “C3” or “scaffold” are cowpea sequences, while sequences starting with “Phvul” are from common bean. The circles on the branches are bootstrap support values from 50 to 100, with the largest circles representing the greatest bootstrap support

**Fig. 9:**
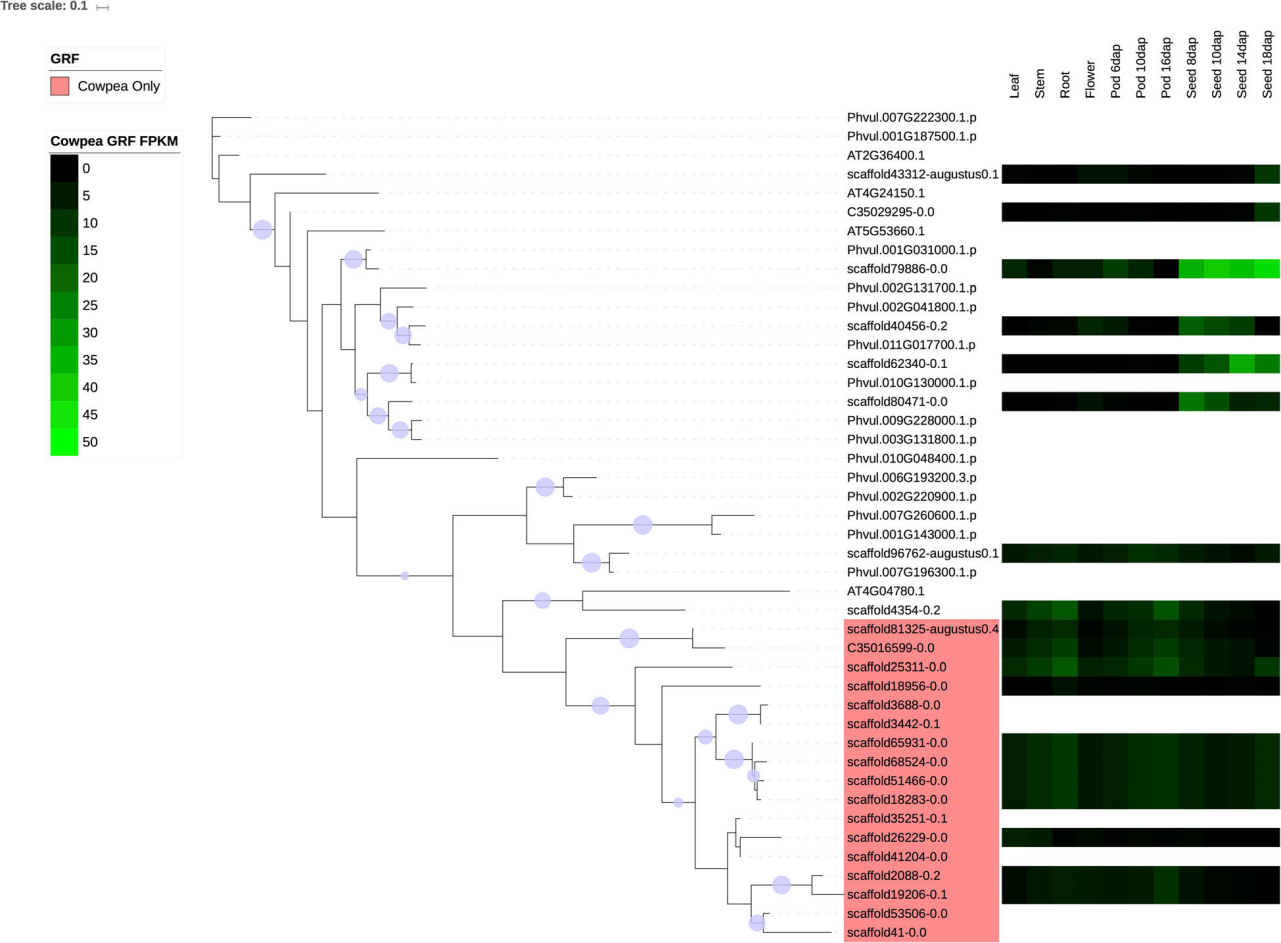
Cowpea and common bean GRF sequences, with predictive heatmaps for cowpea TFs based on FPKM expression values from cowpea transcriptome data on VuGEA [43]. This tree was generated using RAxML [62] with 100 bootstrap values with the optimal amino acid substitution model automatically chosen in RAxML (i.e., the PROTGAMMAAUTO option). Sequences starting with “C3” or “scaffold” are cowpea sequences, while sequences starting with “Phvul” are from common bean. The circles on the branches are bootstrap support values from 50 to 100, with the largest circles representing the greatest bootstrap support

**Fig. 10:**
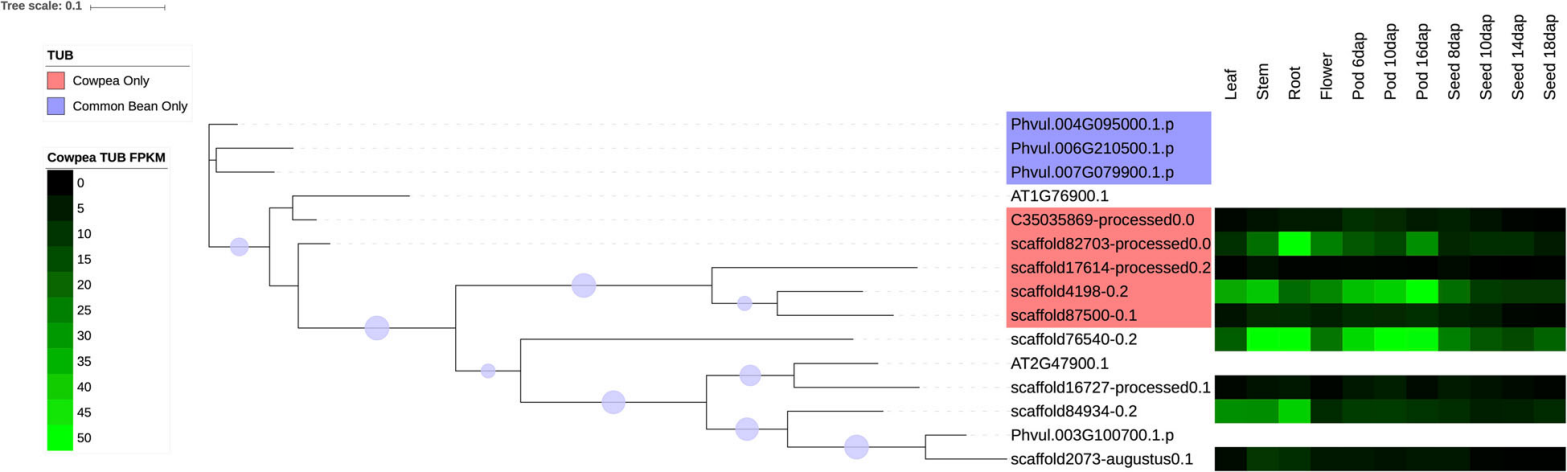
Cowpea and common bean TUB sequences, with predictive heatmaps for cowpea TFs based on FPKM expression values from cowpea transcriptome data on VuGEA [43]. This tree was generated using RAxML [62] with 100 bootstrap values with the optimal amino acid substitution model automatically chosen in RAxML (i.e., the PROTGAMMAAUTO option). Sequences starting with “C3” or “scaffold” are cowpea sequences, while sequences starting with “Phvul” are from common bean. The circles on the branches are bootstrap support values from 50 to 100, with the largest circles representing the greatest bootstrap support

**Fig. 11:**
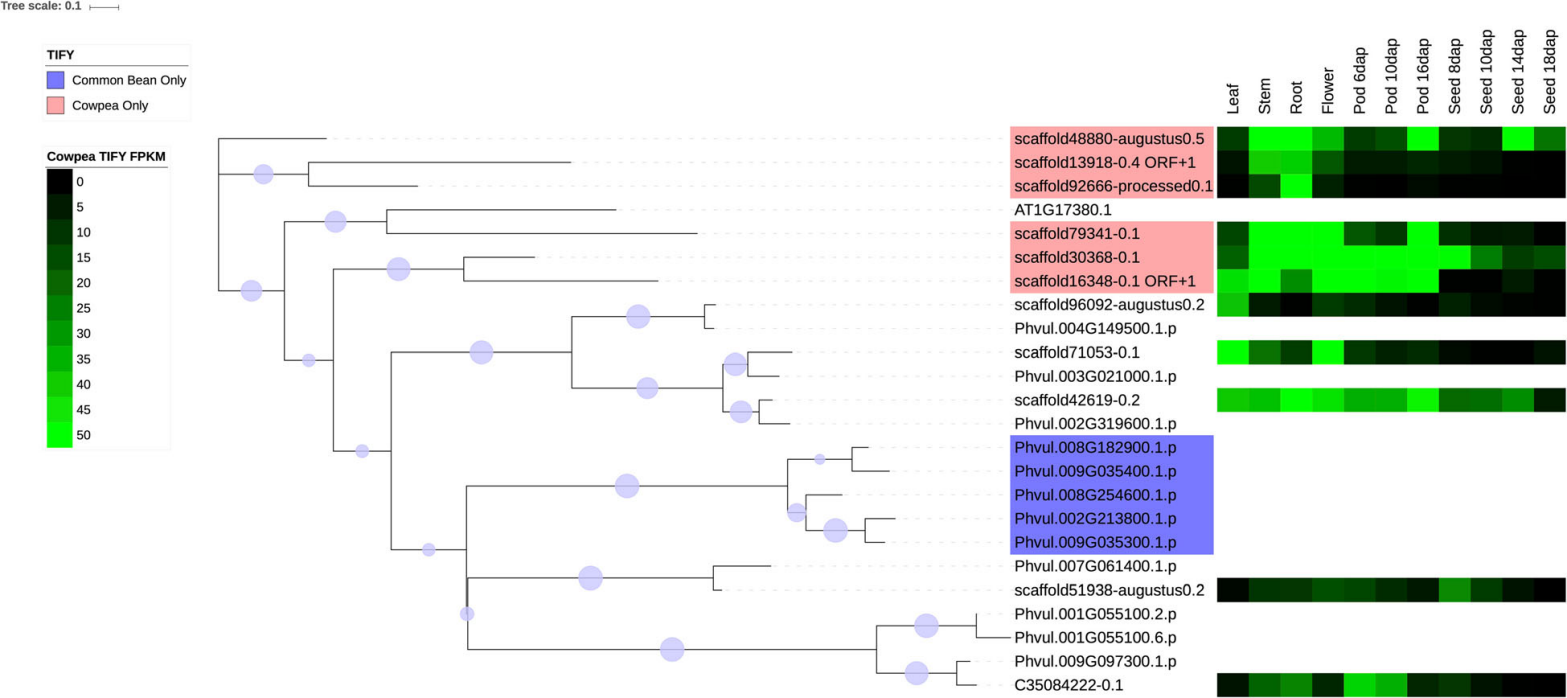
Cowpea and common bean tify sequences, with predictive heatmaps for cowpea TFs based on FPKM expression values from cowpea transcriptome data on VuGEA [43]. This tree was generated using RAxML [62] with 100 bootstrap values with the optimal amino acid substitution model automatically chosen in RAxML (i.e., the PROTGAMMAAUTO option). Sequences starting with “C3” or “scaffold” are cowpea sequences, while sequences starting with “Phvul” are from common bean. The circles on the branches are bootstrap support values from 50 to 100, with the largest circles representing the greatest bootstrap support

Several families were chosen for phylogenetic comparison between cowpea and common bean: the NAC family (Figs. 4 and 6), selected due to its roles in many cellular processes, including development and stress response, two families in which the number of cowpea genes present are under-represented compared to common bean (tify and B3), two families in which the sizes of the gene families are similar in the two species (BES/BZR (Brassinosteroid insensitive 1 (BRI1)-ethyl methanesulfonate (EMS)-suppressor / Brassinazole Resistant) and CCAAT-HAP5), two families in which the sizes of the family are larger (over-represented) relative to common bean (mitochondrial transcription termination factor (mTERF) and TUBBY (TUB)), and two families in which the size of the gene family in cowpea is significantly larger (strongly over-represented) relative to common bean (GRF and Aux/IAA) (see Figs. 7, 8, 9, 10 and 11, Additional files 5, 6 and 7). Here it is important to note that when only genes and not gene models are counted, BES, B3 and CCAAT-HAP5 are under-represented in cowpea compared to common bean, and that mTERF, tify, TUBBY, GRF, NAC and Aux/IAA are over-represented compared to common bean (Additional file 2b). Overall, it was found that cowpea and common bean were more likely to differ in families that are known for having roles in development and stress response.

Figure 6 shows the phylogenetic tree of the NAC family of common bean. In comparison to the NAC family of cowpea (see Fig. 4), three key differences can be found. First, there are five members of group Ic in cowpea, while there are nine members of that same clade in common bean. Second, there are six members of Group II in cowpea as opposed to eight in common bean. Finally, Group IVa has one member in cowpea and four members in common bean (Fig. 6). As stated in the Discussion below, the differences in group Ic and II may be due to possible differences in how cowpea and common bean use NAC TFs in processes such as development and stress response. The differences between cowpea and common bean group IVa seem to indicate a difference in cell cycle control between cowpea and common bean [75].

Figures 7, 8, 9, 10, 11 shows the phylogenetic trees of B3, Aux/IAA, GRF, TUB and tify families. In the phylogenic trees for each family, there are groups of sequences that are unique to cowpea, and in the case of TUB and tify, there are also groups that are unique to common bean. These phylogenetic differences occur in families that have roles in growth and development, as well as in stress response, and thus reflect possible differences in growth, developmental and stress response mechanisms between the two legumes. For example, the B3 family, being a part of the auxin, gibberellic acid (GA) and abscisic acid (ABA) pathways [76], is important in seed development [75; 76] and dehydration response [77].

Additional files 5, 6 and 7 show the phylogenetic trees of the BES/BZR, CCAAT-HAP5, and mTERF families for cowpea and common bean. Cowpea and common bean are similar in phylogenetic organization for these families. The functions of the BES/BZR, CCAAT-HAP5 and mTERF families significantly differ from each other. The BES/BZR family is important in brassinosteroid signaling, which regulates stem cell quiescence in root stem cells [78, 79]. CCAAT-HAP5 TFs, also known as NF-Y subunit C (NF-YC), regulate light-mediated development [80]. The mTERF family has roles in regulating organelle gene expression for a variety of processes [81]. The phylogenetic similarities between cowpea and common bean in these families suggest that a diverse array of cowpea TF families is similarly organized to their counterparts in common bean.

### Expression analyses

To find the stage of development and location of each cowpea TF and TAP sequence in this study, a BLASTN search was performed against the VuGEA database [41] to find for each cowpea TF and TAP in this study, the cowpea transcript with strongest homology (Additional file 8). In the B3, Aux/IAA, GRF, TUB and tify families (Figs. 7, 8, 9, 10 and 11), where cowpea and common bean had different phylogenetic organizations, cowpea TFs were somewhat more likely to express in roots and in pods; in pods, expression showed a slight tendency to be stronger 16 days after planting. In these families, the cowpea TFs that showed stronger expression patterns in roots and pods were more likely to be in groups unique to cowpea. In families where cowpea and common bean show similar organization, by contrast (Additional files 5, 6 and 7), cowpea TFs and TAPs show their strongest expression in varying tissues and phases of development. While these results seem to suggest differences in nutrient gathering, root development and pod development between cowpea and common bean, further study is clearly needed to examine these potential differences.

## Discussion

The availability of a draft genome for cowpea providing 67X coverage [27] has permitted a detailed characterization of the TF and TAP gene families in the genome. While other studies [37, 43] have attempted to identify the repertoire of these regulatory factors in cowpea, prior studies were compromised either by incomplete datasets or by methods that yielded an incomplete repertoire. Our results show that almost all of the known TF and TAP families recognized in other higher plants are represented in the cowpea genome. Cowpea contains a complement of TFs and TAPs that is two thirds as many as its close relative common bean and about one third as many as soybean. Interestingly, when the TF and TAP families in cowpea, common bean and soybean are compared as percentages of their respective total complement, the TFs and TAPs in cowpea are distributed in a manner that is significantly different from common bean and soybean.

This contrasts with expectations given the close relationships between cowpea and other legumes, as well as the strong syntenic relationships between cowpea and soybean [82], between common bean and soybean [83, 84], and between *Vigna* and *Phaseolus* species [85]. However, the discovery by Vascoconcelos et al. (2015) [86] of chromosomal rearrangements between cowpea and common bean, including translocations and duplications, is worth noting.

In the present study, 4416 sequences (from 4408 genes) encoding 5460 TF and TAP encoding domains were found in cowpea, a significant contrast to the 2485 TFs and TAPs found in an analysis of the cowpea transcriptome [44]. This could be due to differences between the annotation in cowpea genome assembly v0.03 [27] and that of the cowpea transcriptome studied in Yao et al. (2016) [44]. Moreover, this study incorporates three separate TF classification pipelines, PlantTFcat [40], iTAK [41] and the TavernaPBS pipeline used in this study [45]; as stated in the Results section, the individual results for each of these pipelines are presented in Additional file 3.

### Phylogenetic analysis of selected TF families

#### AP2-EREBP

The AP2-EREBP family has been investigated in many plants, from *Arabidopsis* and rice [64, 69, 70], tobacco [34], maize [87], and soybean [88], to tomato [89] and cotton [90]. Some of these studies placed AP2, ERF and RAV into the same phylogenetic tree [69, 87], while some others exclusively investigate the ERF family [63; 88], or separate the ERF family from the AP2 and RAV families [90]. Two different classification schemes have emerged, one in which the ERF family is separated into twelve groups (i.e., DREB A1-A6 and ERF B1-B6) [69], and the other in which the ERF family is separated into groups I-X, VI-L and Xb-L [64]. Sharoni et al. (2011) [70] separates the AP2-EREBP into two trees, ERF and AP2/RAV. In this study, the AP2, ERF and RAV families were placed in the same tree (Figs. 2 and 3).

When the twelve-group DREB/ERF classification [69, 70] was employed we found that many of the groups in cowpea are polyphyletic. Dietz et al. (2010) [69] reported some polyphyletic groups in the ERF family of *Arabidopsis*, but not to the extent observed in cowpea. These polyphyletic groups were not found in the ERF family in soybean [88] or rice [70].

When the Nakano et al. (2006) [64] classification scheme was applied, the cowpea ERF family separates more completely into distinct groups, with the exception that group Xb-L appears to be absent in cowpea, and that one group X sequence, Os02g52670.1, does not group with the rest of group X (Fig. 3). The phylogenetic trees presented in Figs. 2 and 3 are similar to that reported by Timko et al. (2008) [26] based upon cowpea GSS data with Groups I-IV forming the DREB clade, and Groups VI, VIII and IX forming the ERF clade. However, Timko et al. (2008) [26] found that Group V ERFs separated between the DREB clade and the ERF clade. In addition, Group IX ERFs separated into two separate clades, with the group VII ERFs between them [26]. In contrast, in this study we observed that the Group V ERFs were in the ERF clade and Groups IX and X are not split by Group VII, but by groups VI and VIII. These differences reflect both the completeness and quality of the sequence data sets and difference in the consistency and accuracy of the alignment tools and phylogenetic methods applied here.

The grouping of the cowpea ERFs into the groups characterized by Nakano et al. (2006) [64] was neater than the grouping into DREB A1-A6 and ERF B1-B6 in Dietz et al. (2010) [69] and Sharoni et al. (2011) [70]. For example, a Group X ERF in rice according to Nakano et al. (2006) [64] was classified as a DREB A5 in Sharoni et al. (2011) [70]; according to Nakano et al. (2006) [64], Group X ERFs represent ERF group B-3 and B-4. Group X in this study was associated with the ERF B-4 clade, consistent with Nakano et al. (2006) [64]. In another example, two DREB sequences from Sharoni et al. (2011) [70] were classified as group VII in Nakano et al. (2006) [64], when the group-VII ERFs in Nakano et al. (2006) [64] were classified as ERF B-2. This could explain why group VII cowpea ERFs grouped with the DREBs in this study (Figs. 2 and 3), unlike in Timko et al. (2008) [26]. The details of the contrast between the classification schemes of Nakano et al. (2006) [64] and Sharoni et al. (2011) [70] are outlined in Additional file 9.

#### NAC family

While methods of classifying NAC TFs into groups vary greatly throughout the literature, regardless of which clustering algorithm one employs, it appears that some legume NAC families are organized differently from other dicots. This difference is evident in the organization of the NAC family in soybean [91] and for rice and *Arabidopsis* [71]. For example, clades Senu5 and Tobacco Elicitor-Responsive NAC protein-like (TERN) (which are VIa and IVc, respectively, in Zhu et al. (2012) [72]), group much farther apart in rice and *Arabidopsis* [71].

When one compares the phylogenetic organization of the cowpea NAC TFs to that observed in other plants using the relative position of each group (closest to furthest from the root of their respective trees) cowpea, not surprisingly, is somewhat closer in organization to the NAC family in Zhu et al. (2012) [72] than to the soybean NAC family in Pinheiro et al. (2009) [90]. Moreover, the cowpea NAC TF family (Fig. 4) differs from common bean in abundance of group Ic, II and IVa NACs (Fig. 6). Our results suggest that even among legumes, the cowpea NAC family may be relatively unique in organization.

Since the NAC family is functionally diverse with regulatory functions ranging from defense against biotic and abiotic stresses, to hormone signaling, and control of reproduction [56] it may not be surprising that plants such as cowpea highly adapted for growth in dry savannah regions would have diversified its NAC family. Thus, the value of our analysis lies in the ability to find those members of this gene family that may confer beneficial growth and development properties onto cowpea that could be exploited for improvement of close relatives like soybean.

#### WRKY family

Both similarities and differences are observed in the WRKY family of cowpea relative to those of other plants. For example, the cowpea WRKY TFs in this study have groups IId and IIe group together as in a Rushton et al. (2010) [60] tree containing soybean, *Arabidopsis thaliana*, rice and poplar. However, in Rushton et al. (2010) [60], Group I N-terminal (NT) and Group I C-terminal (CT) group together, whereas these groups are distant in the cowpea WRKY TFs from this study (Fig. 5). In Li et al. (2012) [74], a phylogenetic tree showing WRKY sequences from *Arabidopsis thaliana*, rice and castor bean shows that like the cowpea WRKY tree in Fig. 5, the WRKYs in Li et al. (2012) [74] are such that Groups I NT and I CT are farther apart. However, groups IIa and IIc in this study group between I and III, whereas in Li et al. (2012) [74], group IIc groups with IIa, b, d and e. When the cowpea WRKY TFs from this study and the WRKYs from Li et al. (2012) [74] are compared from closest to furthest from the root of their respective trees, the following orders are found for this study: Group I NT - > IIa - > IIc - > III - > IIb - > I CT - > IId - > IIe, compared to that of Li et al. (2012) [74]: Group III - > I NT - > IIb - > IIa - > IId - > IIe - > IIc - > I CT. These findings suggest WRKY TF family organization in cowpea is unique.

### Phylogenetic comparison between cowpea and common bean

Overall, TF families that regulate a wide variety of processes in cowpea and common bean are similar in their phylogenetic organization. This is to be expected since cowpea and common bean are close together in the Millettoid (i.e., Phaseoloid) clade of legumes [92]. The BES/BZR, CCAAT-HAP5, and mTERF families in cowpea were all similar in phylogenetic organization to common bean. Thus if a TF family in cowpea is similar in phylogenetic organization but larger in size than in common bean, it may be possible that there are instances of gene duplication (without whole-genome duplication) in that TF family in cowpea; likewise a TF family that is larger in common bean but phylogenetically similar to cowpea may show instances of gene duplication for that TF family in common bean. Instances of such processes have been known to occur throughout plant evolution [30; 93], and they may lead to the presence of sequences with new functions [93].

Among the families analyzed, the B3, Aux/IAA, GRF, TUB, tify and NAC families in cowpea showed some differences in phylogenetic organization from their counterparts in common bean (Figs. 3, 5 and 6). It is possible that different forms of duplication on a small scale are involved in these families. These phylogenetic differences occur in families that have roles in growth and development, as well as in stress response, and thus reflect possible differences in growth, developmental and stress response mechanisms between the two legumes.

#### The B3, GRF, aux/IAA, TUB and tify families

The B3, GRF, Aux/IAA, TUB and tify families have roles in stress response, or in growth and development. As stated earlier, the B3 family has roles in the ABA and GA pathways [76] and in seed development [76, 94] and response to dehydration [77]. The GRF family is associated with a wide array of growth and developmental processes including leaf growth and floral organ development [95, 96], but the Aux/IAA is associated with tolerance to stress [97, 98]. However, it is important to note that Aux/IAA TFs can also be involved in repressing stomatal development in seedlings [99] and root development [100]. The presence of a sequence that is predicted to express most strongly in roots in a group of the Aux/IAA family that is unique to cowpea (Fig. 8) suggests that cowpea root development evolved differently from common bean. The TUB family may have roles in response to abscisic acid (ABA), and thus may be important in stress response [101]. Moreover, experimental studies by Yulong et al. (2016) [101] and Lai et al. (2004) [102] suggest that TUB transcription factors have roles in germinating seedlings. The tify family may have roles in the jasmonic acid (JA) stress response pathway [103]. Since clades exist in these families (with the exception of B3) that are unique to cowpea (Figs. 8, 9, 10 and 11), it is possible that cowpea is evolving to have genes with new functions in growth, development and stress response. Common bean, having expansions in clades of the B3, TUB and tify families (Figs. 7, 10 and 11), may also be evolving to have new genes in these processes, albeit in different ways than in cowpea.

#### NAC

As stated in the Results section, a comparison between the cowpea NACs in Fig. 4 and the common bean NACs in Fig. 6 shows that unlike in cowpea, common bean has a larger clade II and Ic. A search of Os05g10620, a group II NAC, on the Rice Functional Genomic Express Database (RiceGE) database at the Salk Institute Genomic Analysis Laboratory (SIGnAL) [104] shows that the Gene Ontology (GO) annotation of Os05g10620 has “development” as a GO term. Given that Zhu et al. (2012) [72] note that group II is noted to be part of the same monophyletic lineage, the under-representation of group II in cowpea compared to common bean suggests differences in regulation of developmental processes between cowpea and common bean.

Group Ic, also called the Secondary wall-associated NAC Domain (SND) clade [72] has roles in secondary wall development [96]. The greater abundance of group Ic NAC genes in common bean (Fig. 6) further suggests differences from cowpea in regulating developmental processes.

Group IVa NAC sequences are involved in cell cycle control [75], and the over-representation of this group in common bean (Fig. 6) compared to cowpea (Fig. 4) suggests gene duplication and thus the possible presence of genes with new functions [93] in cell cycle control in common bean.

The availability of whole genomic data for cowpea, a non-model legume with significant importance in the developing world, is a significant step forward in orphan legume research. Placing value on this genome sequence by characterization of the various gene families of TFs and TAPs, their organization and phylogenetic relationships, will facilitate future comparative analysis and development of strategies for controlling growth, differentiation, and abiotic and biotic stress resistances.

Overall, our analysis revealed that cowpea, like many of its diploid relatives within the Leguminoseae show gene contents similar to other diploid dicotyledonous plants, and that cowpea, despite having fewer TFs and TAPs in number, has genes coding for almost all of the TF and TAP families in other plants. In many cases the phylogenetic organization of the TF and TAP families in cowpea mimicked their counterparts in common bean, whether or not the number of members of certain TF families in cowpea were significantly different in size compared to their counterparts in common bean. However, aspects of the TF and TAP families of cowpea are unique in composition and organization when compared with its evolutionarily close relatives, common bean and soybean. The functional relevance of these variations can now be explored in greater detail, particularly with regards to growth and developmental processes as well as response to stress.

## Conclusions

We used a computational approach employing three different predictive pipelines to data mine the recently release cowpea genome and identified over 4400 genes representing 136 different TF and TAP families. The availability of detailed information on the coding capacity of the cowpea genome and in particular the various TF and TAP gene families will facilitate future comparative analysis and development of strategies for controlling growth, differentiation, and abiotic and biotic stress resistances of cowpea. By comparison to other closely related legumes we also provide a starting point for additional comparative evolutionary studies.

## Methods

### Genomic data sources and analysis

The analysis presented here used a genomic assembly from cowpea genotype IT97K-499-35 that was an updated version (version 0.03) [27] of the draft assembly (version 0.02) previously described by Pottorff et al. (2012) [105]. The updated version used for this work included one long-insert paired end library (5 kb), which improved the scaffold lengths [27]. Cowpea genome v0.03 is available for BLAST searches and sequence retrieval [106]. All Perl, Bioperl and Bash scripts used in this study are custom scripts that we developed (Additional file 1).

For exon detection and translation of the cowpea assembly v0.03 [27] to protein two approaches were used, one in which the low-complexity regions were masked via AUGUSTUS [51, 52] before using the MAKER annotation pipeline [50], and one that was simply annotated via MAKER and then processed through AUGUSTUS (Stephen M. Turner, personal communication, October 29, 2013).

The soybean genome [31] version Wm82.a2.v1 and the *Phaseolus vulgaris* genome version 2.1 [48, 107] were downloaded from Phytozome v11 [108, 109].

Since two approaches were used for exon detection and translation to proteins as mentioned above, two different versions of the set of protein and nucleotide sequences in the cowpea genome Version 0.03 were produced. These versions were combined into a non-redundant set of sequences using a custom BioPerl script that we designed to make sure that only sequences with unique amino acid compositions were included in the set (Additional file 1).

In addition, to prepare all cowpea, common bean and soybean nucleotide sequences for searches using the TavernaPBS [45] TF classification pipeline, which required amino acid sequences, we used a Bioperl script that we developed to perform a six-frame translation of every nucleotide sequence.

The characterization of TFs and TAPs in cowpea involved identifying TFs and TAPs at the amino acid and the transcript level. In a separate analysis, TFs and TAPs were identified in the raw cowpea genome assembly (the assembly produced prior to exon detection and translation); this was done in order for a comparison with protein and transcript assemblies.

### Classification of transcription factors and transcriptionally active proteins

Transcription factors (TFs) and transcriptionally active proteins (TAPs) were identified by using three approaches: the PlantTFcat pipeline [40], the iTAK pipeline [41], and a TavernaPBS [45] workflow (see Additional file 1) that uses HMMER 3 [110] for Hidden Markov models and PFAM [111] for finding protein domains. TF and TAP classification was done using the rules for membership in each of 136 TF and TAP families, which include 111 different TF and TAP families described by Lang et al. (2010) [44] with only minor adjustments, as well as 43 additional TF and TAP families described in PlantTFcat and iTAK, eighteen of which were synonymous with the 111 TF and TAP families in Lang et al. (2010) [44].

Since both proteins and transcripts were investigated, and three pipelines were used, reducing the sequences to a non-redundant set needed to be done using a Perl script and a Bash script that we developed. Here, the Perl script was used to sort sequences into families with the aid of a text file that not only listed all the TF/TAP families, but took into account the synonyms that different databases used for the same family. The Bash script was developed to follow the following procedure: first, if the protein was present, it was accepted into the set. If not, then translated transcripts from PlantTFcat were accepted. If the PlantTFcat transcript for a certain open reading frame (ORF) (e.g., +2) was present and that same transcript with the same ORF was present in a set of sequences found by iTAK, the latter transcript was eliminated. Otherwise, the iTAK transcript was accepted into the sequence set. Then, if the iTAK transcript for a certain ORF (e.g., +2) was present and that same transcript with the same ORF was present in a set of sequences found by the TavernaPBS pipeline, the latter transcript was eliminated. Otherwise, the transcript was accepted. PlantTFcat was given higher preference than iTAK due to the comprehensive nature of the InterPro database [112] that PlantTFcat uses [40]. iTAK was given higher preference than the TavernaPBS pipeline because the TF and TR classification scheme in iTAK was developed as a consensus between PlantTFDB [37] and PlnTFDB [35, 36] and was developed more recently than the TavernaPBS pipeline based on Lang et al. (2010) [44].

When none of these pipelines identified TFs of a certain family in cowpea, FASTA version 36.3.8e [54] was used to search the raw cowpea genome for the closest homologs of that family, with an E-value of 1e-3. Common bean members of the TF families not found in cowpea were used as queries in these searches.

MEME (Multiple Em for Motif Elicitation) version 4.11.2 [113] was used to identify non-overlapping motifs in each sequence and to aid in curating each TF and TAP family. In motif discovery, an E-value threshold of 0.001, a motif width range of 8–200 amino acids, the “any repeat” (anr) mode active, and maximum number of motifs of 100 was used. We designed two custom scripts that use MEME motif data to curate the sequences, adding each motif in the order that they appear in the sequence: a BioPerl script to parse the MEME data into a table containing sequence name, motif start and end coordinates, and motif sequence; and the other script, a Perl script to use that table to create a curated sequence consisting of the MEME motifs for that sequence in order of start coordinate.

We applied the identical approach to analyzing the TF and TAP contents of common bean and soybean using the soybean and common bean genomes. When comparing common bean sequences for comparison with cowpea sequences, the redundant sequences within the common bean sequence set for a family were removed using the same custom BioPerl script that we designed to remove redundant sequences in the cowpea genome.

### Multiple sequence alignment of TF sequences

Multiple sequence alignment was done using MAFFT version 7.245 [61]. Any duplicate sequence or sequence that covered less than 30% of the alignment was eliminated, and columns that consisted of at least 90% gaps were removed; this was done using trimAl v1.2 [114]. After this, the TF family being analyzed had sequences with 95% similarity or more removed using a custom BioPerl script that we designed, and was re-aligned. This cycle was repeated until the number of sequences and number of alignment columns stabilized.

### Phylogenetic analysis

Phylogenetic trees were generated using RAxML version 8.2.9 [62] with 100 bootstrap replicates. The trees seen in this study were generated using Interactive Tree of Life (iTOL) version 3.3.3 [115]. The trees and their associated alignment files can be found on TreeBASE under the accession 21,817 (http://purl.org/phylo/treebase/phylows/study/TB2:S21817?x-access-code=bc32e308174e24a9e5938101a324a744&format=html) [116].

### Analysis of AP2-EREBP, NAC and WRKY families

For the AP2-EREBP family, selected sequences from each clade of the *Arabidopsis* AP2-EREBP superfamily analyzed in Dietz et al. (2010) [69] and from each clade of the rice AP2-EREBP superfamily analyzed in Sharoni et al. (2011) [70] were used to determine the clades to which cowpea AP2-EREBP sequences belonged, and the AP2-EREBP phylogenetic organization scheme from Dietz et al. (2010) [69] and Sharoni et al. (2011) [70] were compared to that from Nakano et al. (2006) [64]. To analyze the NAC family, sequences from each clade of the NAC family analyzed by Zhu et al. (2012) [72] were used to classify cowpea NAC sequences into clades. To analyze the WRKY family, sequences from each clade of the WRKY family analyzed by Li et al. (2012) [74] were used to classify the cowpea WRKY sequences into clades.

### Expression analysis

All cowpea TF sequences were searched via BLASTN (E-value = 1e-5) against VuGEA [43] to find for each cowpea TF in the cowpea genome, its cowpea transcript with strongest homology in VuGEA, as well as the FPKM expression for that transcript. Upon downloading the FPKM data, we developed custom Perl scripts to link each cowpea TF and TAP to the FPKM expression of the transcript to which it has the greatest similarity. We developed and used a custom Bioperl script to parse the BLASTN search result files from VuGEA, and we designed and used custom Perl scripts to construct heatmaps of the FPKM data, as well as a table of the data showing the cowpea gene, gene model, transcript from VuGEA with strongest homology, and corresponding FPKM values. This table is available in Additional file 8.

## Additional files

Additional file 1: The TavernaPBS workflow used to classify TFs and TAPs in cowpea, common bean and soybean. This workflow was adapted to work in a SLURM environment. Figure adapted from an image of the workflow used in TavernaPBS [45]). This image was generated using Taverna 2.5.0 [46]. The Bioperl scripts and bash scripts and their purposes are also given. (DOC 484 kb)

Additional file 2: Numbers of cowpea TFs, TAPs and TRs compared to common bean and soybean. The comparisons are made with respect to percentage of TF repertoires, raw number and percentage of total protein coding sequences. (XLS 133 kb)

Additional file 3: Cowpea TFs, TAPs and TRs in the A) PlantTFcat, B) iTAK and C) TavernaPBS pipelines. For each pipeline, the numbers shown are from the protein, transcript and raw cowpea assemblies. For A), the cowpea TFs and TRs in VuGEA, which used the PlantTFcat pipeline [40], are presented for comparison. (XLS 76 kb)

Additional file 4: Comparisons of cowpea TF families to their counterparts in common bean and soybean. These comparisons are made with respect to: a) percentage of TF repertoires, b) raw number of TFs, and c) percentage of total protein-coding genes. (JPEG 3544 kb)

Additional file 5: Cowpea and common bean BES_BZR sequences, with predictive heatmaps based on FPKM expression values from cowpea transcriptome data on VuGEA [43]. This tree was generated using RAxML [62] with 100 bootstrap values with the optimal amino acid substitution model automatically chosen in RAxML (i.e., the PROTGAMMAAUTO option). Sequences starting with “C3” or “scaffold” are cowpea sequences, while sequences starting with “Phvul” are from common bean. The circles on the branches are bootstrap support values from 50 to 100, with the largest circles representing the greatest bootstrap support. (JPEG 484 kb)

Additional file 6: Cowpea and common bean CCAAT-HAP5 sequences, with predictive heatmaps based on FPKM expression values from cowpea transcriptome data on VuGEA [43]. This tree was generated using RAxML [62] with 100 bootstrap values with the optimal amino acid substitution model automatically chosen in RAxML (i.e., the PROTGAMMAAUTO option). Sequences starting with “C3” or “scaffold” are cowpea sequences, while sequences starting with “Phvul” are from common bean. The circles on the branches are bootstrap support values from 50 to 100, with the largest circles representing the greatest bootstrap support. (JPEG 954 kb)

Additional file 7: Cowpea and common bean mTERF sequences, with predictive heatmaps based on FPKM expression values from cowpea transcriptome data on VuGEA [43]. This tree was generated using RAxML [62] with 100 bootstrap values with the optimal amino acid substitution model automatically chosen in RAxML (i.e., the PROTGAMMAAUTO option). Sequences starting with “C3” or “scaffold” are cowpea sequences, while sequences starting with “Phvul” are from common bean. The circles on the branches are bootstrap support values from 50 to 100, with the largest circles representing the greatest bootstrap support. (JPEG 2395 kb)

Additional file 8: Cowpea TFs and TAPs with corresponding transcripts in VuGEA, FPKM values, GO annotations, and gene families. This data shows a prediction of where and when cowpea TFs and TAPs express most strongly. (XLS 1840 kb)

Additional file 9: Two different groupings of *Arabidopsis thaliana* and rice (*Oryza sativa*) ERF sequences. The two schemes are the I-X grouping used in Nakano et al. (2006) [64], and DREB A1-A6 / ERF B1-B6 grouping from Dietz et al. (2010) [69] and Sharoni et al. (2011) [70]. (XLS 34 kb)

## Abbreviations

AP2/ERF-ERF: ERF - Ethylene Response Factor
ABA: Abscisic acid
ABI3/VP1: Abscisic Acid Insensitive 3 - Viviparous1
ABTB: Ankyrin Broad Complex, tramtrack and bric a brac
Anr: Any repeat
AP2: APETALA2
AP2-EREBP: APETALA2-Ethylene Responsive Element Binding Protein
ARF: Auxin Response Factor
ATAF2: Arabidopsis transcription activation factor 2
BES/BZR: Brassinosteroid insensitive 1 (BRI1)-ethyl methanesulfonate (EMS)-suppressor / Brassinazole resistant bHLH Basic helix-loop-helix
BLASTN: Basic Local Alignment and Search Tool Nucleotide
bZIP: Basic leucine zipper domain
CCAAT-Dr1: Down regulator of transcription 1
CCAAT-HAP3: CCAAT motif, Heme-associated protein 3
CCAAT-HAP5: CCAAT motif, Heme-associated protein 5
CCHC(Zn): Zinc finger, CCHC-type (CysCysHisCys)
CRs: Chromatin Remodelers
CT: C-terminal
CW-Zn-B3_VAL: CW-like zinc finger, B3, VP1/ABI3-Like (CysTrp)
DREB: Dehydration-responsive element (DRE)-binding
ERF: ERF - Ethylene Response Factor
ESTs: Expressed sequence tags
FAO: Food and Agriculture Organization
GA: Gibberellic acid
GAII: Genome Analyzer II
GO: Gene Ontology
GRF: Growth-Regulating Factor
GSR: Gene space reads (or use GSS for gene space sequences)
HMMs: Hidden Markov Models
iTOL: Interactive Tree of Life
JA: Jasmonic acid
JmjC-ARID: Jumonji C-terminal - AT-rich interaction domain
MADS: Minichromosome maintenance deficient (MCM1), AGAMOUS, DEFICIENS, and serum response factor (SRF)
MAFFT: Multiple Alignment using Fast Fourier Transform
Mb: Megabase
MEME: Multiple Em for Motif Elicitation
mTERF: Mitochondrial transcription termination factor
MYB: Myeloblastosis
MYB-HB-like: MYB Homeobox like
MYB-related: Myeloblastosis-related
NF-X1: Nuclear factor, X-box binding 1
NF-YB: Nuclear factor Y subunit B
NF-YC: NF-Y subunit C
NF-YC: Nuclear factor Y subunit C
NT: N-terminal
ONAC: *Oryza sativa* NAC
ORF: Open reading frame
PBS: Portable Batch System
PcG_FIE: Polycomb Group Fertilization-Independent Endosperm
PHD: Plant Homeodomain
Phvul: *Phaseolus vulgaris*
PlantTAPDB: Plant Transcription Associated Protein Database
PlantTFDB: Plant Transcription Factor Database
PlnTFDB: Plant Transcription Factor Database
RAV: Related to ABI3/VP1
RAxML: Randomized Axelerated Maximum Likelihood
RF-X: Regulatory factor X
RiceGE: Rice Functional Genomic Express Database
Senu5: Senescence Upregulated 5
SIGnAL: Salk Institute Genomic Analysis Laboratory
SLURM: Simple Linux Utility for Resource Management
SND: Secondary Wall-Associated NAC Domain
SOH1: Suppressor of hyper-recombination 1 (hpr1)
TAPs: Transcriptionally Active Proteins / Transcription Associated Proteins
TEA: Transcriptional enhancer activator
TERN: Tobacco Elicitor-Responsive NAC protein-like
TFs: Transcription Factors
TRAF: Tumor necrosis factor receptor-associated factor
TRs: Transcriptional Regulators
TUB: TUBBY
VuGEA: *Vigna unguiculata* Gene Expression Atlas
zn-clus: Zn(2)-Cys(6) binuclear cluster domain
