# Supplementary material for: A compendium of transcription factor and Transcriptionally active protein coding gene families in cowpea (Vigna unguiculata L.)
Source: BMC Genomics. 2017 Nov 22;18:898. doi: 10.1186/s12864-017-4306-1 (PMC5700742; doi:10.1186/s12864-017-4306-1)
Supplement: Supplementary file 1 — The TavernaPBS workflow used to classify TFs and TAPs in cowpea, common bean and soybean. This workflow was adapted to work in a SLURM environment. Figure adapted from an image of the workflow used in TavernaPBS [45]). This image was generated using Taverna 2.5.0 [46]. The Bioperl scripts and bash scripts and their purposes are also given. (DOC 484 kb) [file 12864_2017_4306_MOESM1_ESM.doc]

**The TavernaPBS TF Classification Pipeline**


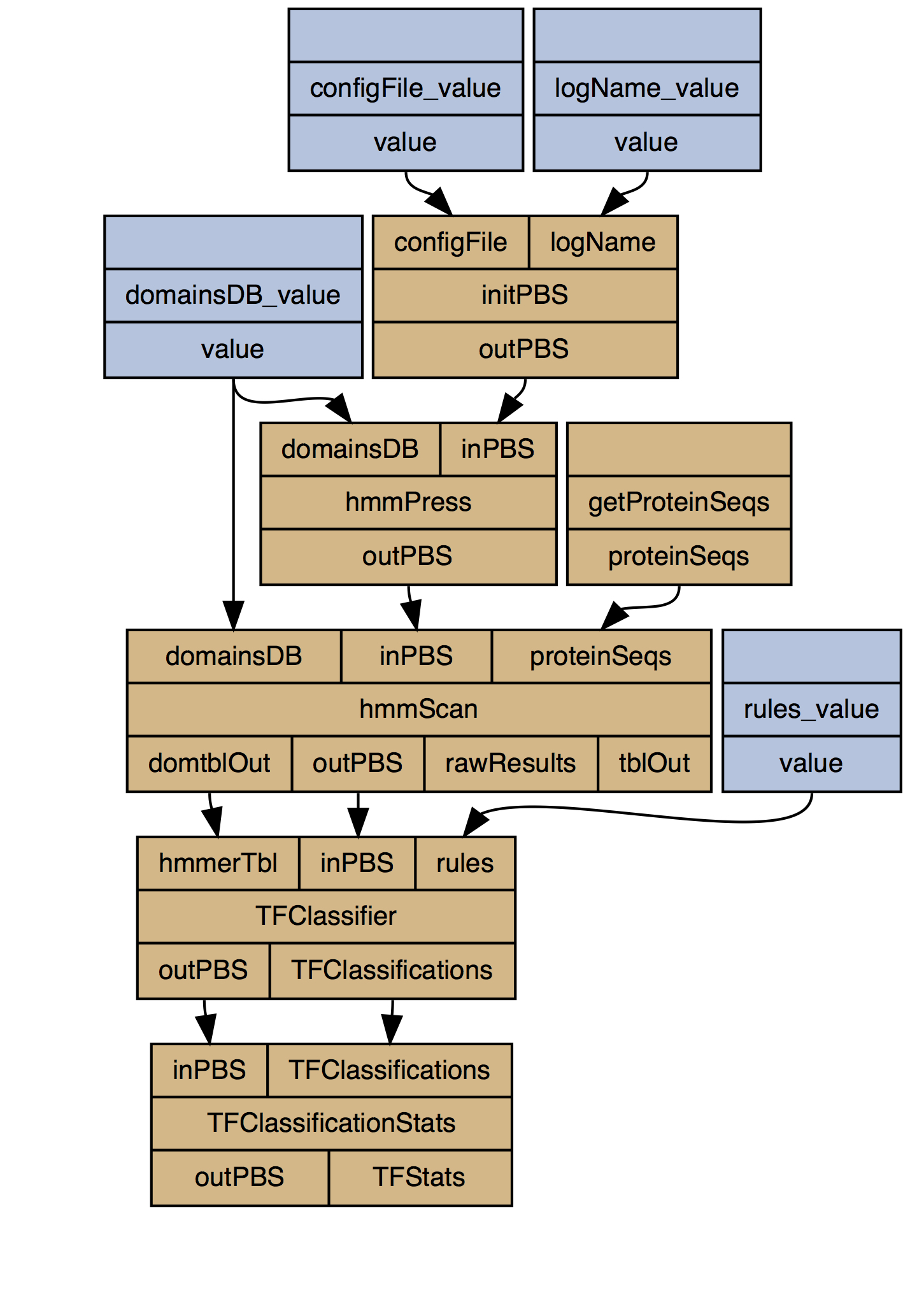


**TF Classification SLURM Script**

#!/bin/bash

#SBATCH --partition=economy

#SBATCH --account=timko-hiseq-analysis

#SBATCH --nodes=1

#SBATCH --ntasks-per-node=4

#SBATCH --mem-per-cpu=4000

#SBATCH --time=48:00:00

cd $SLURM_SUBMIT_DIR

for aa in *.fasta

do

/home/vam5g/hmmer-3.1b2-linux-intel-ia32/src/hmmpress -f /home/vam5g/TFClassification/domainsDB.hmm

/home/vam5g/hmmer-3.1b2-linux-intel-ia32/src/hmmscan -o $aa.raw_results.txt --tblout $aa.tblout.txt --domtblout $aa.domtblout.txt --max --cpu 4 -E 1e-3 --domE 1e-3 /home/vam5g/TFClassification/domainsDB.hmm $aa

/home/vam5g/TFClassification/TFRulesClassifier.pl /home/vam5g/TFClassification/TFRules.txt $aa.domtblout.txt > $aa.TFClassifications.txt

/home/vam5g/TFClassification/TFRulesClassificationStats.pl $aa.TFClassifications.txt > $aa.TFClassifications.stats

done

The code for tf-classifier_slurm.sh , a Bash script we designed for adapting the TavernaPBS pipeline for the SLURM cluster.

**Eliminating Redundant Sequences**

#!/usr/bin/perl

use 5.010;

use strict;

use Bio::SeqIO;

my %unique;

my $file = $ARGV[0];

my $seqio = Bio::SeqIO->new(-file => $file, -format => "fasta");

my $outseq = Bio::SeqIO->new(-file => ">$file.unique.txt", -format => "fasta");

while(my $seqs = $seqio->next_seq) {

my $id = $seqs->display_id;

my $seq = $seqs->seq;

unless(exists($unique{$seq})) {

$outseq->write_seq($seqs);

$unique{$seq} +=1;

}

}

The code for unique-sequences.pl , a BioPerl script we designed for ensuring that only unique amino acid sequences are included in the data set used for analyses.

**DNA Translation in Six Frames**

#!/usr/local/bin/perl

use strict;

use warnings;

use lib '/Users/vikrammisra/.cpan/build/BioPerl-1.007001-1';

use Bio::SeqIO;

use Bio::Seq;

use Bio::DB::Fasta;

use Bio::PrimarySeqI;

my $input_file = $ARGV[0];

my $seqdb = Bio::DB::Fasta->new( $input_file );

my @ids = $seqdb->get_all_primary_ids;

my $dna = '';

for ( my $ind = 0; $ind < scalar @ids; $ind++ ) {

my $seqname = $ids[$ind];

chomp $seqname;

$dna = $seqdb->seq($seqname);

if ($dna =~ /[NnXx]/ ) {

$dna =~ s/[NnXx]/\?/;

}

my $revseq = Bio::Seq->new(-seq=>$dna)->revcom;

my $prot = Bio::Seq->new(-seq=>$dna)->translate->seq;

print `echo \Q>$seqname\_ORF_0F\E >> $input_file.aa.fasta `;

print `echo $prot >> $input_file.aa.fasta`;

$prot = Bio::Seq->new(-seq=>$dna)->translate(-frame => 1)->seq;

# $outprot = ">" . $seqname . "\n" . $prot . "\n";

# print `echo ">$seqname" >> proteins_1f.fasta`;

print `echo \Q>$seqname\_ORF_1F\E >> $input_file.aa.fasta `;

print `echo $prot >> $input_file.aa.fasta`;

$prot = Bio::Seq->new(-seq=>$dna)->translate(-frame => 2)->seq;

# $outprot = ">" . $seqname . "\n" . $prot . "\n";

print `echo \Q>$seqname\_ORF_2F\E >> $input_file.aa.fasta `;

print `echo $prot >> $input_file.aa.fasta`;

$prot = $revseq->translate->seq;

# $outprot = ">" . $seqname . "\n" . $prot . "\n";

print `echo \Q>$seqname\_ORF_0R\E >> $input_file.aa.fasta `;

print `echo $prot >> $input_file.aa.fasta`;

$prot = $revseq->translate(-frame => 1)->seq;

# $outprot = ">" . $seqname . "\n" . $prot . "\n";

print `echo \Q>$seqname\_ORF_1R\E >> $input_file.aa.fasta `;

print `echo $prot >> $input_file.aa.fasta`;

$prot = $revseq->translate(-frame => 2)->seq;

# $outprot = ">" . $seqname . "\n" . $prot . "\n";

print `echo \Q>$seqname\_ORF_2R\E >> $input_file.aa.fasta `;

print `echo $prot >> $input_file.aa.fasta`;

}

The code for dna-translator_6frame.pl , a BioPerl script we designed to translate nucleotide sequences in six frames.

**Separation into TF and TAP Families**

#!/usr/bin/perl

use 5.018;

# Remember to substitute the forward slash for underscore character in the PlantTFcat or other file

# before you begin.

use strict;

use warnings;

open NAMES, $ARGV[0] or die "Error in ARGV 0: $!"; #List of TF Family Names from a database such as PlantTFcat

open TOSEARCH, $ARGV[1] or die "Error in ARGV 1: $!"; # Data file with TF classifications from that database for sequences studied

print "What organism? ";

my $organism = <STDIN>;

chomp($organism);

print "AA, CDS, or Transcript? ";

my $mode = <STDIN>;

chomp($mode);

my @namelist = ();

while (<NAMES>) {

my $name = $_;

chomp($name);

push(@namelist, $name);

}

close NAMES;

my @lines_to_search = ();

while (<TOSEARCH>) {

my $line = $_;

push @lines_to_search, $line;

}

my $nombre;

foreach $nombre (@namelist) {

my $outname = $organism . "_" . $mode . "_" . $nombre . "_lines.txt";

open(my $out, '>', $outname) or die "Cannot open $outname: $!";

my @matches = grep(/$nombre/, @lines_to_search);

print $out join "\n", @matches;

close $out;

}

close TOSEARCH;

The code for family_lines-from-list.pl , a Perl script we designed to separate TF sequence names into text files, each file having the name of the TF family in which the sequence is a member.

**Sorting into TF Families, Accounting for Different Names (Synonyms) that Databases Use for the Same Family**

#!/usr/bin/perl

use strict;

use warnings;

use 5.012;

open DBASE, $ARGV[0] or die "Error in ARGV 0: $!";

my $word;

my $row;

my $col;

my @synonyms = ();

while (<DBASE>) {

chomp;

push @synonyms, [ split /\t/ ];

}

my $elem;

my $outname;

foreach $row (0..@synonyms-1) {

foreach $col (0..@{$synonyms[$row]}-1) {

$elem = $synonyms[$row][$col];

$outname = $synonyms[$row][0];

print `echo *$elem*unique.txt`;

print `cat *$elem*unique.txt >> cowpea-$outname-names.txt`;

print `./unique-lines.pl cowpea-$outname-names.txt`;

}

say "finished " . $outname;

}

close DBASE;

The code for tf-sorter.pl , a perl script we designed to sort TFs into their respective families, taking into account the synonyms that multiple databases have for the same family. For example, if one file of TFs has the name CCAAT_HAP2, and another file of TFs has the name NF-YA, the sequences from these files are sorted into a file with the name CCAAT_HAP2. Please note that this script is to be used after every TF sequence name is first sorted into its corresponding gene family for each pipeline and then stored in a text file bearing the name of that gene family. For example, all sequences belonging to the family “B3-Domain” from PlantTFcat should be stored in a file containing the name “B3-Domain”.

**Eliminate Redundant Lines**

#!/usr/bin/perl

use strict;

use warnings;

open TOOBIG, $ARGV[0] or die "Can't open: $!";

open CLEANED, ">" . $ARGV[0] . "_unique.txt" or die "Can't write: $!";

my $stored;

my @unique;

while (<TOOBIG>) {

my $nombre = $_;

my $count = 0;

foreach $stored (@unique) {

if ($nombre eq $stored) {

$count += 1;

}

}

if ($count == 0) {

print CLEANED $nombre;

push @unique, $nombre;

}

}

close TOOBIG;

close CLEANED;

Code for the unique-lines.pl script, a Perl script we designed to eliminate redundant lines from a text file. The script tf-sorter.pl uses this script and needs to be in the same folder as unique-lines.pl .

**TF Families and Synonyms**

A20-like

Alfin-like

AP2_EREBP AP2_ERF-AP2 AP2_ERF-ERF AP2_ERF-RAV RAV AP2-EREBP

ARF B3-ARF

Argonaute PAZ-Argonaute

ARID ARID-HMG

LOB AS2_LOB AS2-LOB

AUX_IAA AUX-IAA

B3 B3-Domain ABI3_VP1

BBR_BPC BBR-BPC GAGA-Binding-like

BED-type_Zn

BES_BZR BES1

bHLH

bHSH

Bromodomain

BSD

BTB-POZ

bZIP bZIP1 bZIP2

C2C2_CO-like C2C2-CO-like

C2C2_Dof C2C2-Dof

C2C2_GATA C2C2-GATA

C2C2_YABBY C2C2-YABBY

C2C2-LSD Znf-LSD

C2H2

C3H

CAMTA CG1-CAMTA

CCAAT_Dr1

CCAAT_HAP2 Hap2_NF-YA NF-YA

CCAAT_HAP3 Hap3_NF-YB NF-YB

CCAAT_HAP5 NF-YC

CCHC_Zn

CHROMO-DOMAIN

Coactivator_p15 TCoAp15 ssDNA-binding-TF

CPP Tesmin

CSD

CW-Zn

CW-Zn-B3_VAL

DBB Znf-B

DBP

DDT

Dicer

DUF246

DUF296

DUF547

DUF632

DUF833

E2F_DP E2F-DP

EIL

FAR FAR1

FHA FHA-SMAD

FYR

GARP_ARR-B_G2 GARP-ARR-B GARP_ARR-B_Myb RR-B-type

GARP_G2-like GARP-G2-like

GeBP

GIF

GNAT

GRAS

GRF C3H-WRC_GRF

HB

HB-KNOX HB_KNOX Homeodomain-TALE-KNOX

HB-BELL Homeodomain-TALE-BEL

HB-HD-ZIP

HB-other Homeodomain-LIKE

HB-PHD Homeodomain-PHD

HB-WOX Homobox-WOX

HD-SAD

HD-ZIP

HD-Zip_bZIP_1

HD-Zip_Halz

HMG

HRT

HSA

HSF HSF-type-DNA-binding

ISWI

IWS1

JmjC

JmjC-ARID

JmjN

Jumonji

KRAB-box

Lambda-DB

LFY

LIM

LUG LisH

MADS MADS-M-type MADS-MIKC MADS-type1

MBF1

MED6

MED7

mTERF

MYB

MYB_SANT

MYB-HB-like

MYB-related

NAC NAM

NF-X1

Nin-like RWP-RK

None Others

NOZZLE NZZ

OFP

PcG_FIE WD40-like

PcG_VEFS

PHD

PLATZ

Pseudo_ARR-B RR-A-type

RB

Rcd1-like

Rel

RF-X

RRN3

Runt

S1Fa-like

SAP

SBP

SET

Sigma70-like

Sin3

Sir2

SNF2 SWI_SNF_SNF2

SOH1

SRS STY-LRP1

SSXT

STAT

SWI_SNF_BAF60b SWI_SNF-BAF60b

SWI_SNF_SWI3 SWI_SNF-SWI3

SWIB-Plus-3

TAZ

Tc-PD

TCP

TEA

TFb2

Tify

TRAF BTB-POZ-MATH ABTB

Trihelix

TTF-type_Zn

TUB TUBBY

ULT

VARL

VOZ

Whirly

WRKY

YEATS

zf_HD zf-HD

The list of synonyms for each family, used as a text file. Synonyms for the same family are separated by a tab.

**TF Classification Pipeline Prioritization**

#!/bin/sh

FILE=$1 # Sequence names with suffixes such as ORF+2

BASE=$2 # Sequence names without suffixes

while read line ; do

if ( ( ( grep -q "^$line$" $FILE ) || ( grep -q "$line.p" $FILE ) ) && ( ( grep -q "$line\_ORF[+-][123]" $FILE ) || ( grep -q "$line\-[012][FR]" $FILE ) || ( grep -q "$line\-[012][FR]" $FILE ) ) ) ; then

grep -e "^$line$" -e "$line.p" $FILE >> $FILE\_sorted.txt

elif ( ( grep -q "^$line$" $FILE ) || ( grep -q "$line.p" $FILE ) ) ; then #Protein

grep -e "^$line$" -e "$line.p" $FILE >> $FILE\_sorted.txt

elif ( grep -q "$line\_ORF[+-][123]" $FILE ) ; then # PlantTFcat

grep -e "$line\_ORF[+-][123]" $FILE >> $FILE\_sorted.txt

elif ( grep -q "$line\-[012][FR]" $FILE ) ; then

if ( ( grep -q "$line\-0F" $FILE ) && ! ( grep -q "$line\_ORF\+1" $FILE ) ) ; then

grep -e "$line\-0F" $FILE >> $FILE\_sorted.txt

elif ( ( grep -q "$line\-1F" $FILE ) && ! ( grep -q "$line\_ORF\+2" $FILE ) ) ; then

grep -e "$line\-1F" $FILE >> $FILE\_sorted.txt

elif ( ( grep -q "$line\-2F" $FILE ) && ! ( grep -q "$line\_ORF\+3" $FILE ) ) ; then

grep -e "$line\-2F" $FILE >> $FILE\_sorted.txt

elif ( ( grep -q "$line\-0R" $FILE ) && ! ( grep -q "$line\_ORF\-1" $FILE ) ) ; then

grep -e "$line\-0R" $FILE >> $FILE\_sorted.txt

elif ( ( grep -q "$line\-1R" $FILE ) && ! ( grep -q "$line\_ORF\-2" $FILE ) ) ; then

grep -e "$line\-1R" $FILE >> $FILE\_sorted.txt

elif ( ( grep -q "$line\-2R" $FILE ) && ! ( grep -q "$line\_ORF\-3" $FILE ) ) ; then

grep -e "$line\-2R" $FILE >> $FILE\_sorted.txt

fi

elif ( grep -q "$line\_ORF\_[012][FR]" $FILE ) ; then

if ( ( grep -q "$line\_ORF\_0F" $FILE ) && ! ( grep -q "$line\-0F" $FILE ) && ! ( grep -q "$line\_ORF\+1" $FILE ) ) ; then

grep -e "$line\_\ORF\_0F" $FILE >> $FILE\_sorted.txt

elif ( ( grep -q "$line\_ORF\_1F" $FILE ) && ! ( grep -q "$line\-1F" $FILE ) && ! ( grep -q "$line\_ORF\+2" $FILE ) ) ; then

grep -e "$line\_\ORF\_1F" $FILE >> $FILE\_sorted.txt

elif ( ( grep -q "$line\_ORF\_2F" $FILE ) && ! ( grep -q "$line\-2F" $FILE ) && ! ( grep -q "$line\_ORF\+3" $FILE ) ) ; then

grep -e "$line\_\ORF\_2F" $FILE >> $FILE\_sorted.txt

elif ( ( grep -q "$line\_ORF\_0R" $FILE ) && ! ( grep -q "$line\-0F" $FILE ) && ! ( grep -q "$line\_ORF\+1" $FILE ) ) ; then

grep -e "$line\_\ORF\_0R" $FILE >> $FILE\_sorted.txt

elif ( ( grep -q "$line\_ORF\_1R" $FILE ) && ! ( grep -q "$line\-1F" $FILE ) && ! ( grep -q "$line\_ORF\+2" $FILE ) ) ; then

grep -e "$line\_\ORF\_1R" $FILE >> $FILE\_sorted.txt

elif ( ( grep -q "$line\_ORF\_2R" $FILE ) && ! ( grep -q "$line\-2F" $FILE ) && ! ( grep -q "$line\_ORF\+3" $FILE ) ) ; then

grep -e "$line\_\ORF\_2R" $FILE >> $FILE\_sorted.txt

fi

fi

done < $BASE

The script for tfdb-heirarchy.sh , a Bash script designed to prioritize TF classification schemes as specified in the Materials and Methods section.

**MEME Results to FASTA Sequence Part 1 – MEME to Data Table**

#!/usr/local/bin/perl

use strict;

use warnings;

use 5.010;

use lib '/Users/vikrammisra/.cpan/build/BioPerl-1.007001';

use Bio::SeqFeatureI;

use Bio::AlignIO;

use Bio::SimpleAlign;

use Bio::Seq;

use Bio::LocatableSeq;

use Bio::SeqFeatureI;

use Bio::FeatureHolderI;

use List::MoreUtils qw(uniq);

use Data::Dumper qw(Dumper);

my $in_name = $ARGV[0];

open INFILE, '<', $in_name or die "Could not read $ARGV[0]: $!\n";

my $in = Bio::AlignIO->new(-file => $in_name, -format => 'meme');

open OUTFILE, '>' . $ARGV[0] . "-table.txt" or die "Could not write: $!\n";

# say OUTFILE "ID\tMotif Start\tMotif End\tAA Sequence";

while ( my $aln = $in->next_aln ) {

foreach my $seqobj ($aln->each_seq()) {

my $table_line = $seqobj->id . "\t" . $seqobj->start() . "\t" . $seqobj->end() . "\t" . $seqobj->seq;

say OUTFILE $table_line;

}

}

close INFILE;

close OUTFILE;

The script for meme-to-table_aa.pl , a BioPerl script designed to take MEME motif results for amino acid sequences (and nucleotide sequences translated into six frames) and make a table of sequence names, motif start and end coordinates and found motif within the query sequence.

**MEME Results to FASTA Sequence Part 2 – Data Table to FASTA**

#!/usr/local/bin/perl

use strict;

use warnings;

use 5.010;

use List::MoreUtils qw(uniq);

open INTABLE, '<' . $ARGV[0] or die "Could not read: $!";

open NEWFASTA, '>' . $ARGV[0] . "-results.fasta" or die "Could not write: $!";

my %starts;

my %startset = ();

my %seqset = ();

my @nameset = ();

while (<INTABLE>) {

my $line = $_;

chomp($line);

my @elements = split "\t", $line;

my $seqid = $elements[0];

my $begin = $elements[1];

my $finish = $elements[2];

my $aaseq = $elements[3];

$startset{$line} = $begin;

$seqset{$line} = $aaseq;

unless ( grep( /$seqid/, @nameset) ) {

push @nameset, $seqid;

}

}

my @sortednames = sort { lc($a) cmp lc($b) } @nameset;

foreach my $id ( @sortednames ) {

say NEWFASTA ">" . $id;

foreach my $startkey ( sort { $startset{$a} <=> $startset{$b} } keys %startset ) {

if ( $startkey =~ /^$id/ ) {

# print NEWFASTA $startkey;

print NEWFASTA $seqset{$startkey};

}

}

print NEWFASTA "\n";

}

close INTABLE;

close NEWFASTA;

The script for memetable_to-fasta.pl , a Perl script designed to join MEME motifs in the order that they appear in the original query sequence, thus producing a curated amino acid sequence.

**Remove Sequences with Greater than 95% Similarity**

#!/usr/bin/perl

use 5.010;

use strict;

use warnings;

use Bio::SeqIO;

use Bio::AlignIO;

unless ( defined $ARGV[0] ) {

die "Cannot open $ARGV[0]: $!";

}

my $str = Bio::AlignIO->new(-file => $ARGV[0], -format => 'fasta');

my $out = Bio::AlignIO->new(-file => ">$ARGV[0].lt95.txt", -format => 'fasta');

my ($aln) = $str->next_aln();

$aln->purge(0.95);

$out->write_aln($aln);

The code for purge95.pl , a BioPerl script we designed to remove sequences that are greater than 95% similar to each other, to ensure an accurate alignment.

**BLAST Parser**

#!/usr/bin/perl

use 5.010;

use strict;

use Bio::SearchIO;

my @conserved;

my $searchio = Bio::SearchIO->new( -format => 'blast',

-file => $ARGV[0] );

while ( my $result = $searchio->next_result() ) {

while( my $hit = $result->next_hit ) {

while( my $hsp = $hit->next_hsp ) {

print $result->query_name, "\t", $hit->name, "\n";

}

}

}

The code for blastparser.pl , a BioPerl script we designed to parse the BLASTN results from VuGEA, retrieving the query and the hit sequence name. Because the hit sequences were already listed from strongest homology to weakest homology, Microsoft Excel was used to take the two-column list of query and hit sequence, andremove redundant values in the query name column, leaving the query sequence name and the corresponding best hit sequence name.

**Retrieve FPKM for a Transcript in VuGEA**

#!/usr/bin/perl

use 5.010;

use strict;

use warnings;

open DATALIST, "<" . $ARGV[0] or die "Error in sequence file: $!";

open NAMELIST, "<" . $ARGV[1] or die "Error in name list: $!";

my %data_entries = ();

while (<DATALIST>) {

my $colone = $_;

my $therest = $_;

chomp($colone);

chomp($therest);

$colone =~ s/\t.*$//;

$therest =~ s/^(.*?)\t//;

$data_entries{$colone} = $therest;

}

close DATALIST;

my %groups = ();

while (<NAMELIST>) {

my $firstcol = $_;

my $secondcol = $_;

chomp($firstcol);

$firstcol =~ s/\t.*$//;

$secondcol =~ s/^(.*?)\t//;

$secondcol =~ s/\t.*$//;

push @{ $groups{$firstcol} }, $secondcol;

}

close NAMELIST;

my @datanames = keys %data_entries;

my @subsets = keys %groups;

#foreach my $tester ( values %data_entries ) {

#}

#foreach my $gridname ( @datanames ) {

foreach my $category ( @subsets ) {

foreach my $gridname ( @datanames ) {

my $outfile = $category . "\\" . "_data.txt";

my @matches = grep /$gridname/, @{ $groups{$category} };

foreach my $val ( @matches ) {

if ( $data_entries{$gridname} =~ /\d/ ) {

my $output_line = $val . "\t" . $data_entries{$gridname};

my $finalform = `echo "$output_line" >> $outfile`;

} else {

next;

}

}

}

}

Code for fpkm-getter_vugea.pl , a Perl script we designed to retrieve the FPKM data for transcripts from VuGEA.

**Preparing Heatmaps for a TF Family**

#!/usr/bin/perl

use 5.010;

use strict;

use warnings;

open DATALIST, "<" . $ARGV[0] or die "Error in sequence file: $!";

open NAMELIST, "<" . $ARGV[1] or die "Error in name list: $!";

my %data_entries = ();

while (<DATALIST>) {

my $colone = $_;

my $therest = $_;

chomp($colone);

chomp($therest);

$colone =~ s/\t.*$//;

$therest =~ s/^(.*?)\t//;

$data_entries{$colone} = $therest;

}

close DATALIST;

my %groups = ();

while (<NAMELIST>) {

my $firstcol = $_;

my $secondcol = $_;

chomp($firstcol);

$firstcol =~ s/\t.*$//;

$secondcol =~ s/^(.*?)\t//;

$secondcol =~ s/\t.*$//;

push @{ $groups{$firstcol} }, $secondcol;

}

close NAMELIST;

my @datanames = keys %data_entries;

my @subsets = keys %groups;

#foreach my $tester ( values %data_entries ) {

#}

#foreach my $gridname ( @datanames ) {

foreach my $category ( @subsets ) {

foreach my $gridname ( @datanames ) {

my $outfile = $category . "\\" . "_data.txt";

my @matches = grep /$gridname/, @{ $groups{$category} };

foreach my $val ( @matches ) {

if ( $data_entries{$gridname} =~ /\d/ ) {

my $output_line = $val . "\t" . $data_entries{$gridname};

my $finalform = `echo "$output_line" >> $outfile`;

} else {

next;

}

}

}

}

Code for search-data-by-group.pl , a Perl script we designed to take a two-column list with the first column being the cowpea genome sequence name and second column being the names of corresponding best-hit transcripts from VuGEA, and then use the VuGEA transcript name to find the FPKM data, and then create a table with the cowpea genome name in place of the VuGEA transcript name. This was done to prepare the FPKM data of a TF family for displaying on a tree in iTOL.

**Making a Table with Gene, Genome Sequence and Data and Annotations for Best-Hit Transcript on VuGEA**

#!/usr/bin/perl

use 5.010;

use strict;

use warnings;

open DATALIST, "<" . $ARGV[0] or die "Error in sequence file: $!";

open NAMELIST, "<" . $ARGV[1] or die "Error in name list: $!";

# Note: In NAMESET, the first column is assumed to be groups whereas the name

# itself is in the second column.

my %data_entries = ();

while (<DATALIST>) {

my $colone = $_;

my $therest = $_;

chomp($colone);

chomp($therest);

$colone =~ s/\t.*$//;

$therest =~ s/^(.*?)\t//;

$data_entries{$colone} = $therest;

# say $colone . "\t" . $therest;

}

close DATALIST;

my %groups = ();

while (<NAMELIST>) {

my $firstcol = $_;

my $secondcol = $_;

chomp($firstcol);

chomp($secondcol);

$firstcol =~ s/\t.*$//;

$secondcol =~ s/^(.*?)\t//;

$secondcol =~ s/\t.*$//;

push @{ $groups{$firstcol} }, $secondcol;

}

close NAMELIST;

my @datanames = keys %data_entries;

my @subsets = keys %groups;

#foreach my $tester ( values %data_entries ) {

# say $tester;

#}

#foreach my $gridname ( @datanames ) {

# foreach my $category ( @subsets ) {

foreach my $category ( @subsets ) {

foreach my $gridname ( @datanames ) {

# my $outfile = $category . "\\" . "_data.txt";

my @matches = grep /$gridname/, @{ $groups{$category} };

foreach my $val ( @matches ) {

# foreach my $val ( @{ $groups{$category} } ) {

# if (( $val =~ /$gridname/ ) && ( $data_entries{$gridname} =~ /\d/ )) {

# if ( ( $val =~ /$gridname/ ) && ( defined $data_entries{$gridname} ) ) {

if ( $data_entries{$gridname} =~ /\d/ ) {

say $category . "\t" . $val . "\t" . $data_entries{$gridname};

# say $output_line;

# my $finalform = `echo "$output_line" >> $outfile`;

} else {

next;

}

}

}

}

Code for annotation-table-prep.pl , a Perl script we designed to take the list of gene families and each member of these families, and then for each member, search against a table of VuGEA data, and then produce a file containing, for each genome sequence name, the best-hit VuGEA transcript, and the GO annotations and FPKM data for that transcript. Here the genome sequence name is the nucleotide sequence used to search against VuGEA, and its name does not contain any suffixes. This same script can then be used to treat the cowpea genome sequence and its data as two columns, and then retrieve the family for that genome sequence from a two-column list with the family as the first column and the genome sequences (which in this list has the suffixes such as “_ORF”).
