# Supplementary figures and images for: A compendium of transcription factor and Transcriptionally active protein coding gene families in cowpea (Vigna unguiculata L.)
Source: BMC Genomics. 2017 Nov 22;18:898. doi: 10.1186/s12864-017-4306-1 (PMC5700742; doi:10.1186/s12864-017-4306-1)

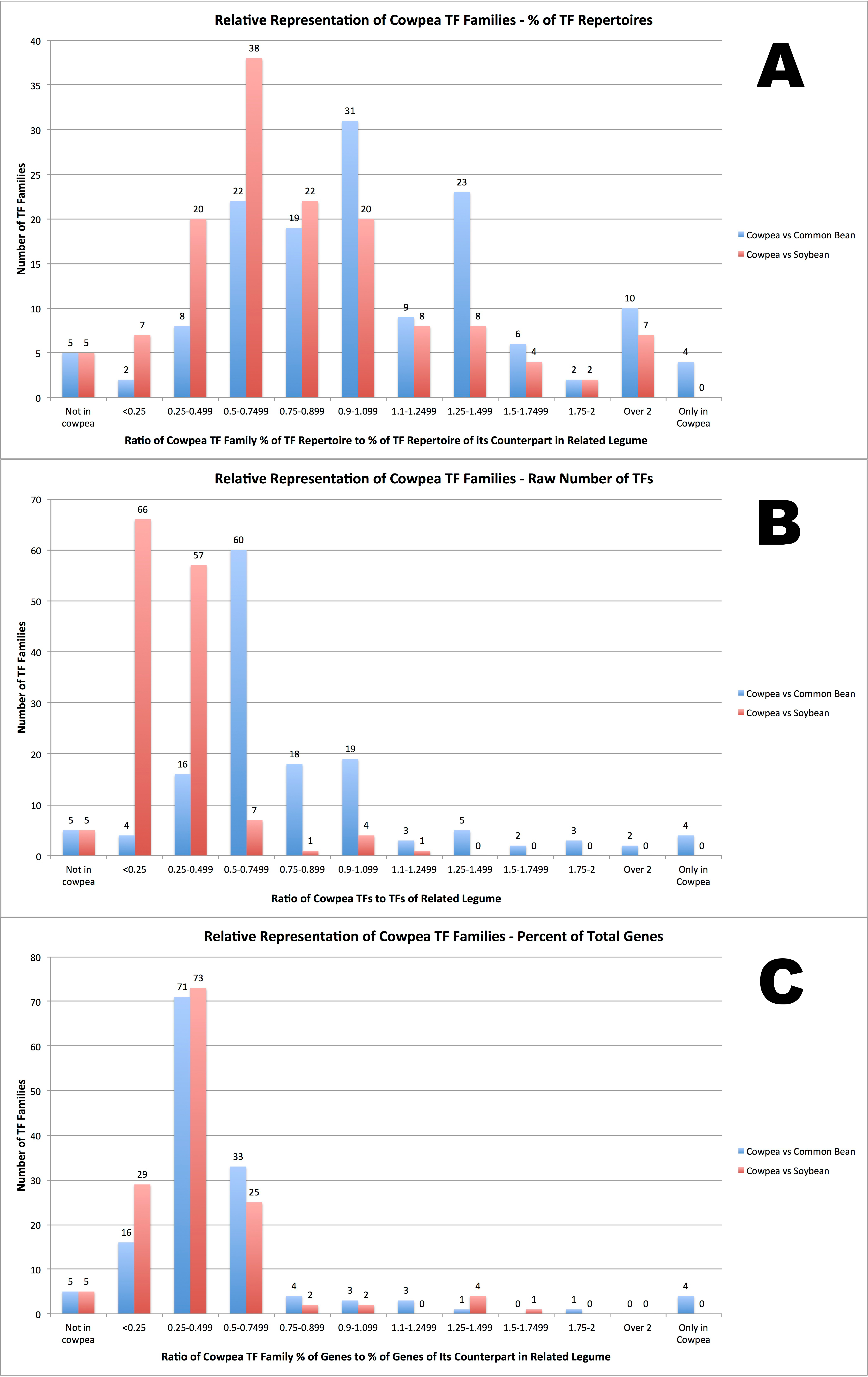

Supplement: Supplementary file 4 — Comparisons of cowpea TF families to their counterparts in common bean and soybean. These comparisons are made with respect to: a) percentage of TF repertoires, b) raw number of TFs, and c) percentage of total protein-coding genes. (JPEG 3544 kb) [file 12864_2017_4306_MOESM4_ESM.jpg]

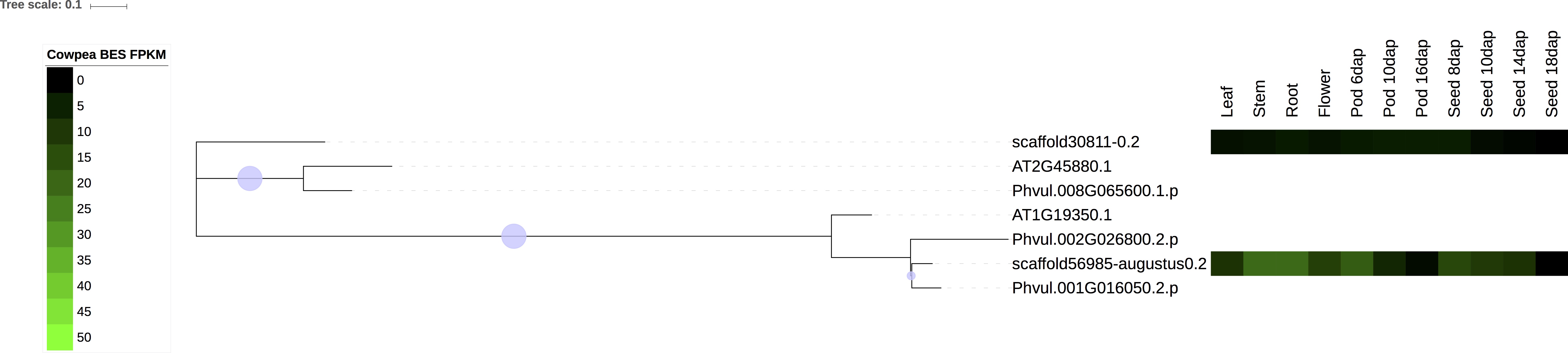

Supplement: Supplementary file 5 — Cowpea and common bean BES_BZR sequences, with predictive heatmaps based on FPKM expression values from cowpea transcriptome data on VuGEA [43]. This tree was generated using RAxML [62] with 100 bootstrap values with the optimal amino acid substitution model automatically chosen in RAxML (i.e., the PROTGAMMAAUTO option). Sequences starting with “C3” or “scaffold” are cowpea sequences, while sequences starting with “Phvul” are from common bean. The circles on the branches are bootstrap support values from 50 to 100, with the largest circles representing the greatest bootstrap support. (JPEG 484 kb) [file 12864_2017_4306_MOESM5_ESM.jpg]

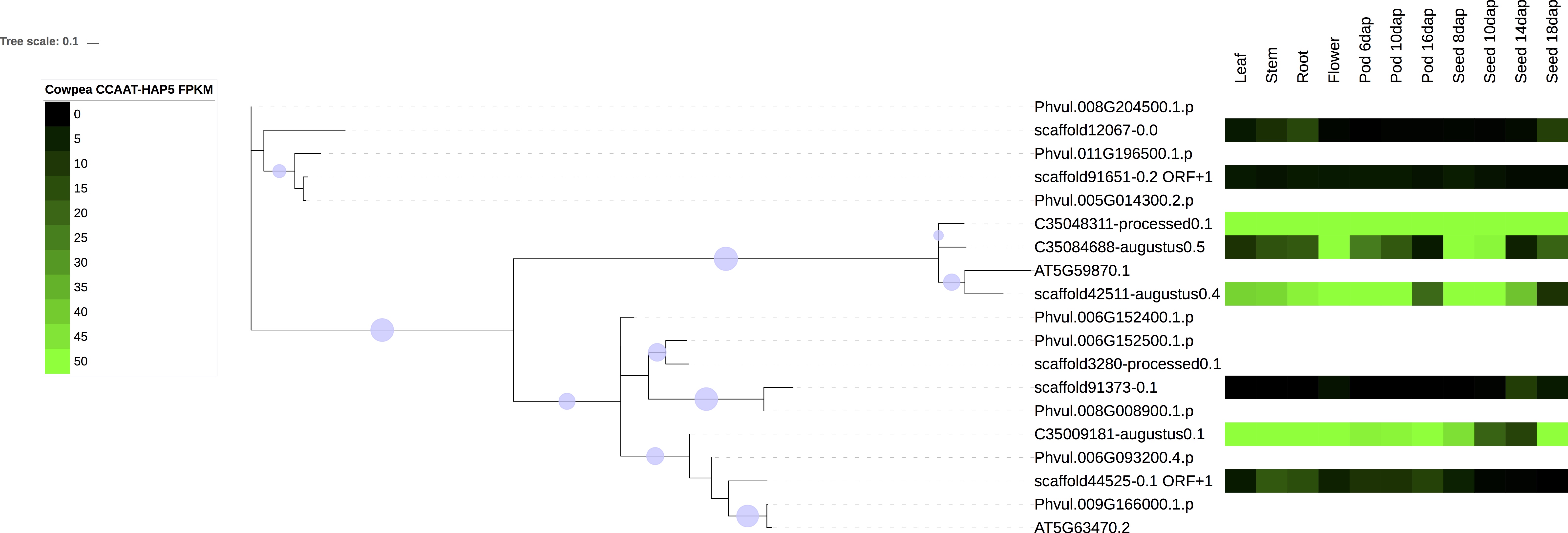

Supplement: Supplementary file 6 — Cowpea and common bean CCAAT-HAP5 sequences, with predictive heatmaps based on FPKM expression values from cowpea transcriptome data on VuGEA [43]. This tree was generated using RAxML [62] with 100 bootstrap values with the optimal amino acid substitution model automatically chosen in RAxML (i.e., the PROTGAMMAAUTO option). Sequences starting with “C3” or “scaffold” are cowpea sequences, while sequences starting with “Phvul” are from common bean. The circles on the branches are bootstrap support values from 50 to 100, with the largest circles representing the greatest bootstrap support. (JPEG 954 kb) [file 12864_2017_4306_MOESM6_ESM.jpg]

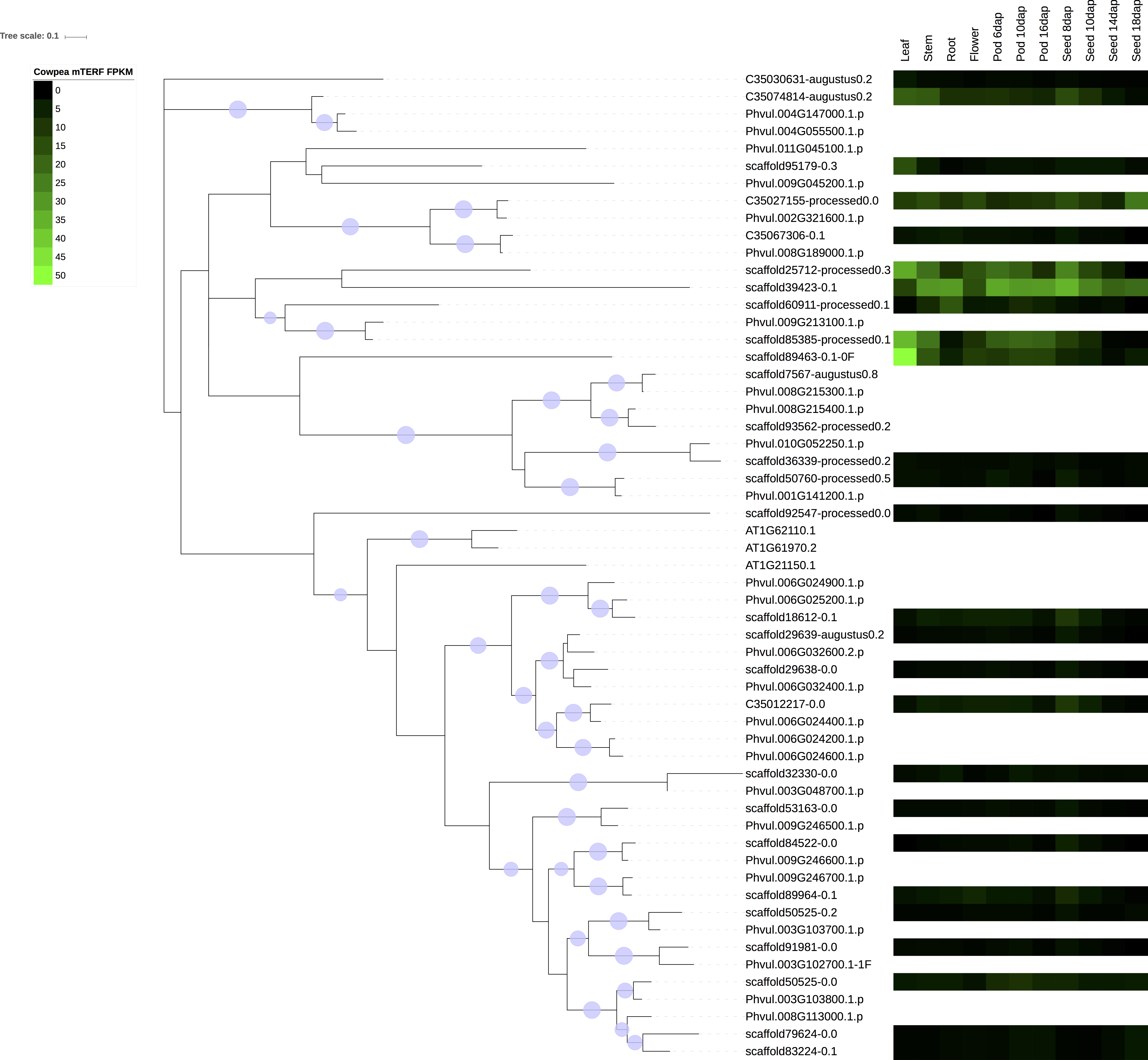

Supplement: Supplementary file 7 — Cowpea and common bean mTERF sequences, with predictive heatmaps based on FPKM expression values from cowpea transcriptome data on VuGEA [43]. This tree was generated using RAxML [62] with 100 bootstrap values with the optimal amino acid substitution model automatically chosen in RAxML (i.e., the PROTGAMMAAUTO option). Sequences starting with “C3” or “scaffold” are cowpea sequences, while sequences starting with “Phvul” are from common bean. The circles on the branches are bootstrap support values from 50 to 100, with the largest circles representing the greatest bootstrap support. (JPEG 2395 kb) [file 12864_2017_4306_MOESM7_ESM.jpg]
